# Memory CD8 T cells mediate severe immunopathology following respiratory syncytial virus infection

**DOI:** 10.1371/journal.ppat.1006810

**Published:** 2018-01-02

**Authors:** Megan E. Schmidt, Cory J. Knudson, Stacey M. Hartwig, Lecia L. Pewe, David K. Meyerholz, Ryan A. Langlois, John T. Harty, Steven M. Varga

**Affiliations:** 1 Interdisciplinary Graduate Program in Immunology, University of Iowa, Iowa City, Iowa, United States of America; 2 Department of Microbiology and Immunology, University of Iowa, Iowa City, Iowa, United States of America; 3 Department of Pathology, University of Iowa, Iowa City, Iowa, United States of America; 4 Department of Microbiology and Immunology, University of Minnesota, Minneapolis, Minnesota, United States of America; 5 Center for Immunology, University of Minnesota, Minneapolis, Minnesota, United States of America; Nationwide Children’s Hospital, UNITED STATES

## Abstract

Memory CD8 T cells can provide protection from re-infection by respiratory viruses such as influenza and SARS. However, the relative contribution of memory CD8 T cells in providing protection against respiratory syncytial virus (RSV) infection is currently unclear. To address this knowledge gap, we utilized a prime-boost immunization approach to induce robust memory CD8 T cell responses in the absence of RSV-specific CD4 T cells and antibodies. Unexpectedly, RSV infection of mice with pre-existing CD8 T cell memory led to exacerbated weight loss, pulmonary disease, and lethal immunopathology. The exacerbated disease in immunized mice was not epitope-dependent and occurred despite a significant reduction in RSV viral titers. In addition, the lethal immunopathology was unique to the context of an RSV infection as mice were protected from a normally lethal challenge with a recombinant influenza virus expressing an RSV epitope. Memory CD8 T cells rapidly produced IFN-γ following RSV infection resulting in elevated protein levels in the lung and periphery. Neutralization of IFN-γ in the respiratory tract reduced morbidity and prevented mortality. These results demonstrate that in contrast to other respiratory viruses, RSV-specific memory CD8 T cells can induce lethal immunopathology despite mediating enhanced viral clearance.

## Author summary

Memory CD8 T cells have been shown to provide protection against many respiratory viruses. However, the ability of memory CD8 T cells to provide protection against RSV has not been extensively examined. Unexpectedly, mice with pre-existing CD8 T cell memory, in the absence of memory CD4 T cells and antibodies, exhibited exacerbated morbidity and mortality following RSV infection. We demonstrate that the immunopathology is the result of early and excessive production of IFN-γ by memory CD8 T cells in the lung. Our research provides important new insight into the mechanisms of how memory T cells induce immunopathology. In addition, our findings serve as an important cautionary tale against the use of epitope-based T cell vaccines against RSV.

## Introduction

Respiratory syncytial virus (RSV) is a major cause of severe disease in young children, the elderly, and immunocompromised populations [1–6]. Furthermore, RSV is the leading cause of infant hospitalizations creating an immense healthcare burden for treatment and prevention [1, 2, 7–11]. There is currently no licensed vaccine for RSV. During a primary RSV infection, the CD8 T cell response is crucial for mediating viral clearance [12, 13]. Depletion of CD8 T cells in mice prior to RSV challenge leads to elevated viral loads, but also ameliorates morbidity [12]. Thus, CD8 T cells contribute to both viral clearance and immunopathology following an acute RSV infection. RSV-specific memory CD8 T cells also contribute to protection from a secondary infection [12]. Antibody-mediated depletion of memory CD8 T cells in RSV-immune mice impairs viral clearance following re-infection as compared to non-treated controls [12]. Thus, vaccines that elicit robust memory CD8 T cell responses may help promote long-lived immunity against RSV.

The induction of neutralizing antibodies remains a primary goal of most RSV vaccines due to their clearly established capacity to reduce the severity of RSV-induced disease [14–17]. In contrast, studies have demonstrated that robust memory CD4 T cell responses can mediate vaccine-enhanced disease following RSV infection [18, 19]. Adoptive transfer of activated effector RSV-specific CD8 T cells, in vitro stimulated T cell lines, or in vitro propagated T cell clones leads to enhanced RSV clearance from the lung following RSV challenge. These effector CD8 T cell transfers were also associated with increased weight loss, indicating that infusion of effector CD8 T cells can induce increased systemic disease [20–23]. However, the role of memory CD8 T cells in providing protection against RSV infection remains unclear. Evaluating the capacity of memory CD8 T cells to mediate protection against RSV infection is critically important because high neutralizing antibody titers alone are insufficient to prevent RSV-induced disease in every individual [14, 24].

Herein, we evaluated the protective capacity of memory CD8 T cells against RSV infection in the absence of RSV-specific CD4 T cell memory and antibodies. We employed a dendritic cell-*Listeria monocytogenes* (DC-LM) prime-boost immunization regimen to induce high magnitude, RSV epitope-specific CD8 T cell responses in naive mice. A similar prime-boost immunization strategy has been shown to elicit protection against other respiratory viruses including influenza A virus (IAV) and severe acute respiratory syndrome coronavirus (SARS-CoV) [25, 26]. DC-LM immunization induced robust memory CD8 T cell responses that reduced viral titers following RSV challenge. However, despite enhanced viral clearance, immunized mice experienced increased pulmonary disease, weight loss, and mortality. Exacerbated disease and mortality was unique to the context of an RSV infection as immunized mice were protected against challenge with a lethal dose of a recombinant IAV expressing an RSV-derived CD8 T cell epitope. The lethal immunopathology observed in immunized mice was caused by rapid and excessive IFN-γ production by memory CD8 T cells in the airways. Our studies reveal that memory CD8 T cells enhance RSV clearance similar to other viral infections, but are unique in that they mediate severe immunopathology caused by the overproduction of IFN-γ.

## Results

### DC-LM immunization induces high-magnitude memory CD8 T cell responses that reduce viral load upon RSV challenge

**Fig 1:**
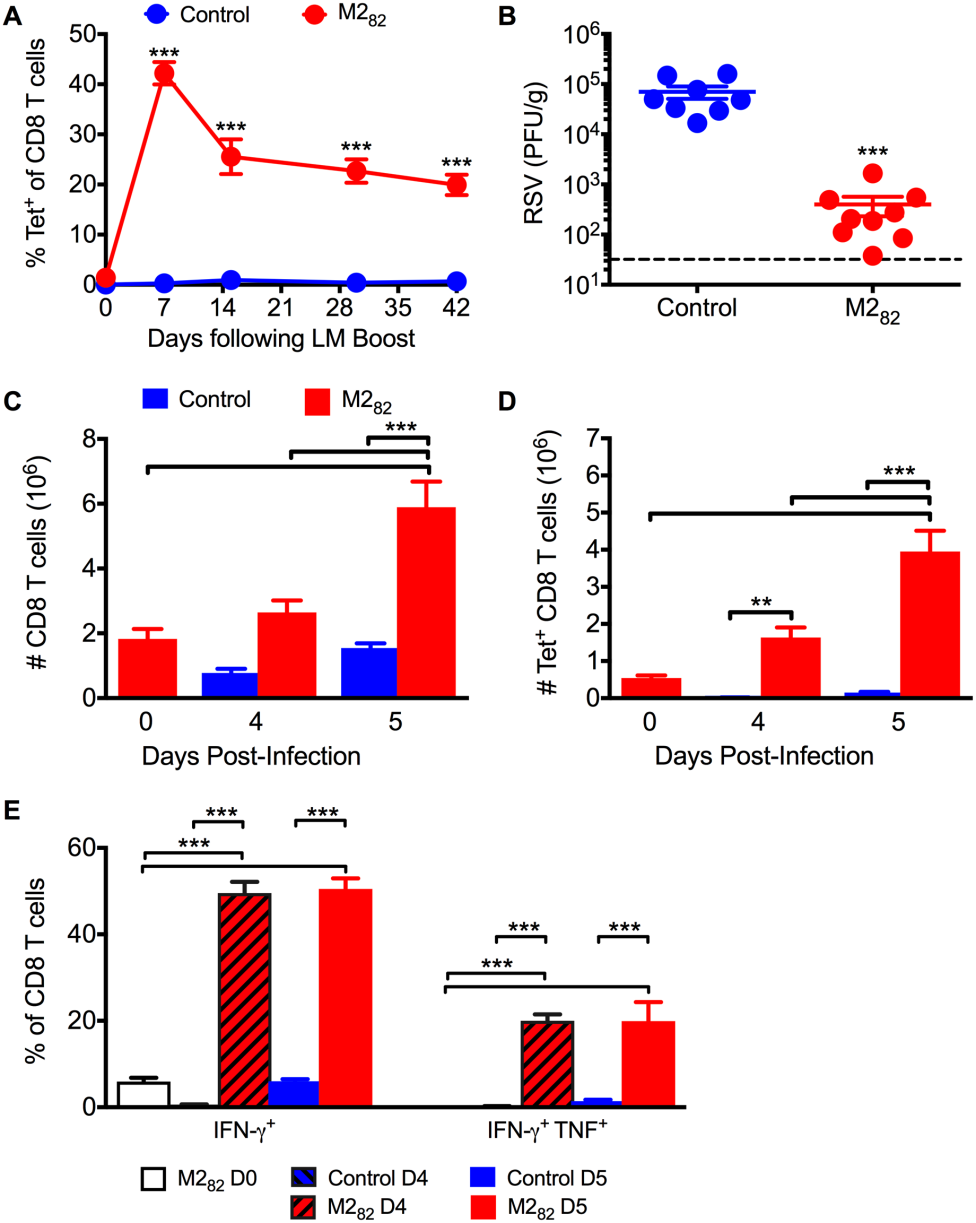
DC-LM immunization induces robust memory CD8 T cell responses that mediate clearance following RSV infection. Naive BALB/c mice were primed with mature DCs coated in M2_82_ peptide and boosted 7 days later with recombinant LM expressing the M2_82_ epitope. Control mice received mature DCs not coated in peptide and were inoculated with LM that did not express an RSV-derived epitope. (A) M2_82_-tetramer^+^ CD8 T cell response was assessed in the PBL following DC-LM prime-boost immunization. (B) RSV titers at day 4 p.i. were determined via plaque assay in the lung. (C) Total CD8 and (D) M2_82_-specific CD8 T cells were quantified in the lungs of immunized mice at days 0, 4, and 5 p.i. (E) Frequency of CD8 T cells in the lung producing either IFN-γ or IFN-γ and TNF after M2_82_-peptide re-stimulation was determined at days 0, 4, and 5 p.i. Data are represented as mean ± SEM of two independent experiments (*n* = 8 mice). Groups were compared using Student’s *t* test for (A, B) and one-way ANOVA in (C-E), ** *p*<0.01, *** *p*<0.001.

Peptide-coated, mature DCs can be utilized to prime a CD8 T cell response that allows for robust secondary expansion following a booster immunization in mice [27]. To induce RSV-specific CD8 T cell memory in the absence of virus-specific CD4 T cells and antibodies, naive mice were immunized with matured splenic DCs loaded with M2_82-90_ (M2_82_) peptide and boosted 7 days later via infection with an attenuated, recombinant LM strain expressing the M2_82_ epitope. A control group without RSV-specific CD8 T cell memory was generated by immunizing mice with DCs not exposed to peptide followed by a boost with an LM that did not express an RSV-derived epitope. DC-LM immunization led to a significant (*p*<0.001) increase in M2_82_-specific CD8 T cell frequencies following the LM booster inoculation within the peripheral blood leukocytes (PBL) compartment as compared to the control group (Fig 1A). Approximately 20% of all CD8 T cells in the PBL were M2_82_-specific at day 42 post-boost (Fig 1A). Immunized mice challenged with RSV exhibited a significant (*p*<0.001) reduction in lung viral titers by day 4 post-infection (p.i.) compared to the control group undergoing a primary RSV infection (Fig 1B). As expected, immunized mice exhibited an increased number of total and M2_82_-specific CD8 T cells in the lung by day 5 p.i. as compared to non-infected M2_82_-immunized controls (Fig 1C and 1D). In addition, there was a greater frequency (*p*<0.001) of IFN-γ^+^ and IFN-γ^+^TNF^+^ CD8 T cells following M2_82_ peptide re-stimulation at days 4 and 5 p.i. as compared to control groups (Fig 1E). Thus, DC-LM immunization elicited robust memory CD8 T cell responses that mediated enhanced viral clearance following RSV challenge.

Pre-existing memory CD8 T cells altered the response of specific cell types following RSV infection (S1 Fig). Both conventional CD4 and regulatory CD4 T cell (Treg) numbers were significantly reduced (*p*<0.05) at days 4 and 5 p.i. in the lung compared to the acute infection control group. Furthermore, the number of monocytes were significantly (*p*<0.05) increased at day 5 p.i. as compared to control mice. These changes were in contrast to eosinophil, neutrophil, and natural killer (NK) cell responses, which remained similar to non-infected M2_82_-immunized mice. Overall, DC-LM immunization induced memory CD8 T cells that altered the magnitude of the subsequent cellular infiltrate and enhanced viral clearance following RSV infection.

### RSV-specific memory CD8 T cells induce immunopathology that is unique to the context of an RSV infection

**Fig 2:**
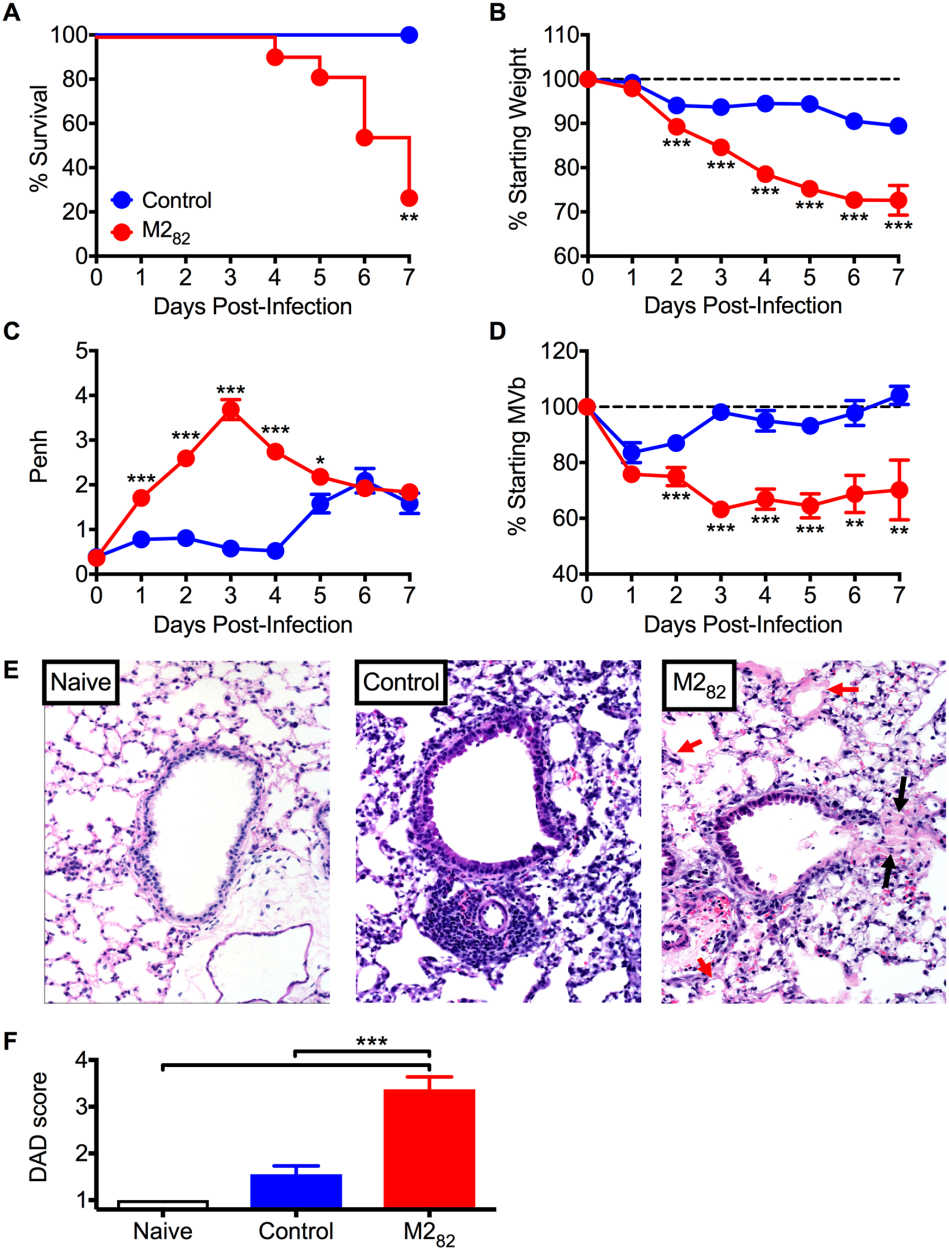
Memory CD8 T cells contribute to lethal pulmonary immunopathology upon RSV challenge. Control- and M2_82_-immunized mice were assessed for (A) survival, (B) weight loss, (C) Penh, and (D) MVb following RSV infection. (E) Lungs from naive, control, and M2_82_ DC-LM-immunized mice were collected at day 5 p.i. and sections were stained for H&E. Representative images were captured at 200X magnification. Black arrows highlight a region of cellular debris and fibrin partially obstructing the alveolar duct. Red arrows highlight multifocal hyaline membranes. (F) Diffuse alveolar damage (DAD) scores were determined on day 5 following infection. Data are represented as mean ± SEM of two independent experiments (*n* = 7 naive mice, *n* = 8 mice for control and M2_82_ groups). Groups were compared using Student’s *t* test for (A-D) and one-way ANOVA in (F), * *p*<0.05, ** *p*<0.01, *** *p*<0.001.

Since DC-LM immunized mice exhibited decreased viral titers, we next determined if they also experienced reduced disease severity. We evaluated weight loss and airway obstruction, both key disease manifestations that can be assessed following RSV infection in mice [18, 28, 29]. Despite enhanced viral control, M2_82_-immunized mice exhibited a significant (*p*<0.01) decrease in survival (Fig 2A). Approximately 40% of fatalities were due to M2_82_-immunized mice naturally succumbing to RSV infection, while 60% were euthanized upon reaching a humane weight loss endpoint. This outcome was both unexpected and unusual since an acute RSV infection is rarely fatal in adult BALB/c mice. M2_82_-immunized mice also exhibited significantly (*p*<0.05) increased weight loss (Fig 2B) and reduced pulmonary function (Fig 2C and 2D). Additionally, we evaluated lungs by histology for evidence of diffuse alveolar damage (DAD), an acute form of lung injury [30, 31]. If severe and extensive enough, DAD is the foundational lesion in the clinical syndrome known as acute respiratory distress syndrome. M2_82_-immunized mice revealed increased (*p*<0.001) histopathological evidence of characteristics associated with early stages of DAD including cellular sloughing and necrosis, alveolar hemorrhage, early cellular infiltrates, and hyaline membrane formation (Fig 2E and 2F and S2 and S3 Figs).

Previous work has demonstrated that the M2_82_-specific CD8 T cell response contributes to the immunopathology associated with an acute RSV infection [32]. Thus, it was unclear if the increased disease severity observed in M2_82_-immunized mice was unique to the M2_82-90_ epitope. To address this possibility, we evaluated mice immunized against the F_85-93_ (F_85_) CD8 T cell epitope following RSV infection [33]. Similar to M2_82_-immunized mice, F_85_ DC-LM immunization induced a high frequency of RSV F_85_-specific memory CD8 T cells that mediated a decrease in lung viral titers at day 4 following RSV challenge (S4A and S4B Fig). In addition, F_85_-immunized mice exhibited increased mortality, weight loss, and pulmonary dysfunction as compared to controls (S4C–S4F Fig). Thus, the severe immunopathology induced by memory CD8 T cells was not specific to either a particular epitope or an RSV protein. We next determined if RSV-specific memory CD8 T cells would also cause increased disease in C57BL/6 mice, as an acute RSV infection in this mouse strain typically causes only mild disease [34]. Therefore, we immunized C57BL/6 mice against the immunodominant M_187-195_ (M_187_) CD8 T cell epitope [35]. DC-LM immunization targeting the M_187_ epitope resulted in approximately 33% M_187_-specific CD8 T cells in the PBL by day 28 post-boost (S5A Fig). Similar to M_82_-immunized BALB/c mice, M_187_-immunized C57BL/6 mice exhibited significantly reduced lung virus titers (*p*<0.001), decreased pulmonary function, and increased weight loss following RSV infection (S5B–S5E Fig). However, in contrast to M2_82_-immunized BALB/c mice, all of the M_187_-immunized C57BL/6 mice survived following RSV infection. This data indicates that M_187_-specific CD8 T cells also contribute to immunopathogenic responses in the C57BL/6 genetic background.

The challenge virus utilized in our study is the RSV A2 strain, which primarily induces a Th1-biased immune response [36]. However, other RSV strains can induce more heterogeneous Th responses. To determine if memory CD8 T cells generated by DC-LM vaccination induce immunopathology independently of the RSV challenge strain, we infected M2_82_-immunized mice with the recombinant RSV A2-line19F strain, which promotes a more Th2-biased immune response than RSV A2 [36]. M2_82_-immunized mice challenged with A2-line19F exhibited mortality, weight loss, and pulmonary dysfunction that was identical to mice challenged with A2 (S6 Fig). These results indicate that M2_82_-specific memory CD8 T cells induce immunopathology independently of the RSV strain utilized for challenge.

**Fig 3:**
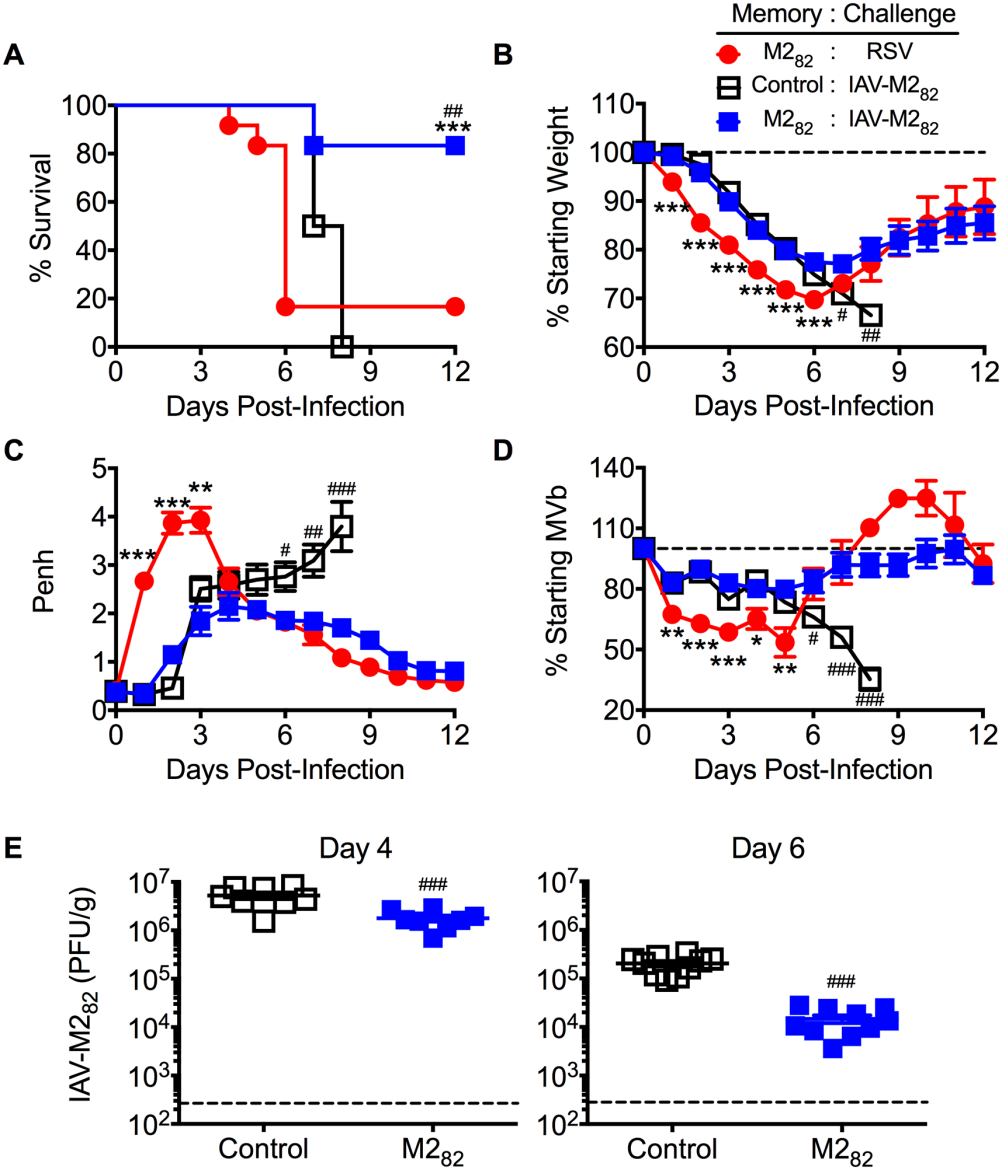
M2_82_-specific CD8 T cell memory protects against lethal heterologous IAV-M2_82_ infection. Control and M2_82_-immunized mice were challenged with either RSV or a 5 LD_50_ dose of IAV-M2_82_ and monitored daily for (A) survival, (B) weight loss, (C) Penh, and (D) MVb. (E) IAV titers in the lungs of IAV-M2_82_-infected M2_82_-immunized mice were determined at day 4 or 6 p.i. via plaque assay. Data are represented as mean ± SEM of two independent experiments (*n* = 12 mice for (A-D), *n* = 8–10 mice for (E)). Statistical comparisons were made using Student’s *t* test in (E) and one-way ANOVA for (A-D), *^/#^ *p*<0.05, **^/##^ *p*<0.01, ***^/###^ *p*<0.001. Asterisks represent statistical significance between M2_82_: RSV and M2_82_: IAV-M2_82_ groups, while pound symbols indicate a difference between Control: IAV-M2_82_ and M2_82_: IAV-M2_82_ groups.

To determine if M2_82_-specific memory CD8 T cells would be pathogenic outside of RSV infection, we challenged M2_82_-immunized mice with a recombinant IAV expressing the M2_82_ epitope (IAV-M2_82_). Slutter et al. recently used a similar DC-LM prime-boost strategy to elicit IAV-specific memory CD8 T cells and demonstrated protection against a subsequent IAV infection [25]. Similar to RSV challenge, immunized mice infected with IAV-M2_82_ exhibited a significant increase in the number of total and M2_82_-specific CD8 T cells in the lung by day 5 p.i. (S7 Fig). IAV-M2_82_ infection also resulted in a significant (*p*<0.05) increase in monocytes and decrease in eosinophils compared to non-infected M2_82_-immunized controls (S7 Fig). CD4 T cell, Treg, neutrophil, and NK cell numbers were not significantly altered in IAV-M2_82_ challenged mice (S7 Fig). As anticipated, all control mice, which do not have virus-specific memory CD8 T cells, succumbed to a lethal IAV-M2_82_ challenge. However, in contrast to the significant (p<0.001) mortality observed in M2_82_-immunized mice following RSV challenge, M2_82_-immunized mice were protected against a lethal IAV-M2_82_ infection (Fig 3A). Pre-existing M2_82_-specific memory CD8 T cells also prevented the prolonged weight loss and pulmonary dysfunction observed in control mice following IAV-M2_82_ infection (Fig 3B–3D). Furthermore, weight loss and pulmonary dysfunction following RSV challenge were significantly (*p*<0.05) increased at early timepoints compared to IAV-M2_82_ infected mice (Fig 3B–3D). In addition, M2_82_-specific memory CD8 T cells induced a significant (*p*<0.001) reduction in IAV titers at both day 4 and day 6 p.i. (Fig 3E). These data indicate that pre-existing RSV M2_82_-specific memory CD8 T cells mediate enhanced IAV-M2_82_ clearance and promote increased survival of mice by preventing prolonged disease. To confirm that memory CD8 T cells would also provide protection without extensive immunopathology to a lower viral inoculum, we challenged M2_82_-immunized mice with a sublethal dose of IAV-M2_82_. Similar to a lethal dose of IAV-M2_82_, M2_82_-immunized mice experienced significantly (*p*<0.05) improved pulmonary function and less weight loss as compared to the control group following a sublethal infection (S8 Fig). Thus, memory CD8 T cells do not promote distinct patterns of disease severity between sublethal and lethal IAV infections. These results indicate that lethal immunopathology associated with a high-magnitude memory CD8 T cell response is unique to the context of an RSV infection.

### M2_82_-specific resident memory CD8 T cells induced by local immunization ameliorate immunopathology following RSV challenge

The DC-LM immunization strategy utilized in our study induced high frequency systemic CD8 T cell memory but resulted in fatal immunopathology following RSV challenge. We hypothesized that a local immunization would promote a large population of antigen-specific resident memory CD8 T cells within the lung that would prevent the immunopathology observed after RSV infection. To evaluate the effects of local immunization, DC-M2_82_-primed mice were either not boosted, given a systemic LM-M2_82_ boost intravenously (i.v.), or given a local IAV-M2_82_ boost intransally (i.n.). Administration of an IAV-M2_82_ boost induced a significant (*p*<0.001) increase in the total number of M2_82_-specific CD8 T cells in the lung prior to RSV challenge as compared to a systemic LM-M2_82_ boost (S9A and S9B Fig). We next utilized intravascular staining to determine the localization of cells within the lung following immunization [37, 38]. The majority of M2_82_-specific CD8 T cells in the lung of DC-M2_82_-primed mice that were either not boosted or boosted systemically with LM-M2_82_ were located within the pulmonary vasculature (S9C Fig). In contrast, greater than 85% of M2_82_-specific CD8 T cells were localized within the lung tissue in IAV-M2_82_-boosted mice, resulting in a significant (*p*<0.001) increase in total number as compared to LM-M2_82_-boosted mice (S9C and S9D Fig). In addition, local boost in the lung with IAV-M2_82_ induced a large frequency of RSV-specific CD8 T cells within the lung tissue expressing both CD69 and CD103, which represent the canonical markers of resident memory CD8 T cells. In contrast, systemic LM-M2_82_ immunization failed to elicit resident memory CD8 T cells within the lung tissue (S9E and S9F Fig).

To determine whether the RSV-specific resident memory CD8 T cells generated by local immunization induce less severe immunopathology than their systemically induced counterparts, DC-M2_82_-primed mice that were either not boosted or boosted with either LM-M2_82_ i.v. or IAV-M2_82_ i.n. were challenged with RSV and monitored for morbidity and mortality. In contrast to a systemic LM-M2_82_ boost, a local boost in the lung with IAV-M2_82_ resulted in 100% survival following RSV challenge (S10A Fig). The IAV-M2_82_ boost also resulted in significantly (*p*<0.05) reduced weight loss and pulmonary dysfunction following RSV infection compared to the LM-M2_82_ boost (S10B–S10D Fig). These results suggest that local prime-boost immunization generates RSV-specific resident memory CD8 T cells that prevent fatal immunopathology and ameliorate disease following RSV challenge.

### Role of perforin and TNF in the induction of immunopathology

We next evaluated the primary antiviral effector functions of memory CD8 T cells to determine their contribution to the immunopathology. The primary pathway of CD8 T cell-mediated cytolysis is through the release of perforin and granzymes [39–41]. Therefore, we evaluated survival and disease severity following RSV infection of perforin-deficient M2_82_-immunized mice. Mice deficient in perforin exhibited accelerated mortality as compared to wild-type (WT) controls (S11A Fig). The kinetics of weight loss and pulmonary dysfunction were similar between WT and perforin-deficient M2_82_-immunized mice following RSV challenge (S11B–S11D Fig). Therefore, perforin is not required to mediate exacerbated disease, but may be necessary to prevent additional mortality.

We also evaluated the role of TNF in immunized mice given its previously identified contribution to immunopathology associated with an acute RSV infection [42]. Antibody-mediated neutralization of TNF in the airways at the time of RSV challenge led to survival of all M2_82_-immunized mice (S12A Fig). Neutralization of TNF significantly (*p*<0.05) reduced both weight loss and pulmonary dysfunction (S12B–S12D Fig). These data illustrate that similar to an acute RSV infection [42], TNF also contributes to the immunopathology associated with memory CD8 T cell responses. Furthermore, TNF neutralization had no significant impact on viral titers at day 4 p.i. (S12E Fig). Assessment of TNF levels at day 2 following RSV challenge revealed similar levels between control- and M2_82_-immunized mice (S12F Fig). TNF levels in the lung were significantly (*p*<0.01) increased in M2_82_-immunized mice at day 4 p.i. as compared to the control group, but no increase was observed in the serum (S12F Fig). However, the overall amount of TNF in both the lung and the serum had decreased by day 4 p.i., the time when immunized mice began to succumb to the RSV infection. Therefore, these data demonstrate that TNF contributes to general inflammation in the lung during both a primary and recall response to RSV infection, but that TNF is necessary for the lethal immunopathology to occur.

### Virus-specific CD8 T cells in the airways rapidly secrete excessive amounts of IFN-γ following RSV infection

**Fig 4:**
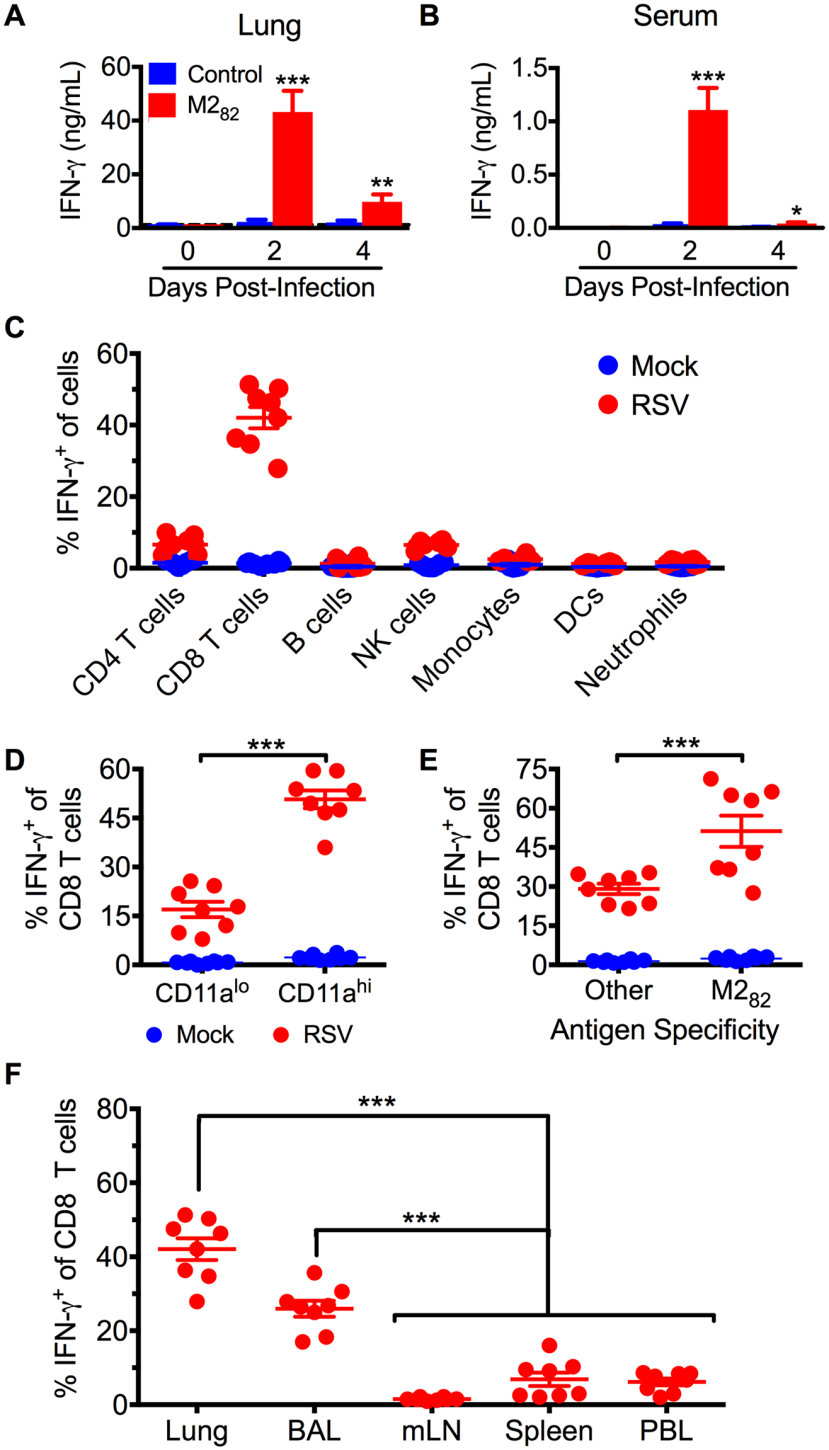
Airway CD8 T cells secrete excessive amounts of IFN-γ upon RSV infection in M2_82_-immunized mice. IFN-γ protein amount was determined in the (A) lung and (B) serum at 0, 2, and 4 days following RSV challenge of immunized mice. (C) DC-LM-immunized mice were treated with 250 μg BFA 6 hours prior to tissue collection. In vivo IFN-γ production was assessed for major leukocyte populations in the lung 2 days following either mock or RSV infection. Frequency of IFN-γ-producing CD8 T cells based upon (D) CD11a expression and (E) M2_82_ epitope specificity 2 days after either mock or RSV infection. (F) Frequency of IFN-γ^+^ CD8 T cells was assessed in the lung, BAL, mLN, spleen, and PBL at day 2 p.i. Data are represented as mean ± SEM of two independent experiments (*n* = 6 for control and *n* = 8 for M2_82_ in (A, B); *n* = 8 for (C-F)). Groups were compared using Student’s *t* test for (A-E) and one-way ANOVA in (F), * *p*<0.05, ** *p*<0.01, *** *p*<0.001.

Due to the accelerated memory CD8 T cell response in DC-LM immunized mice, we speculated that the early inflammatory cytokine milieu would be distinct from an acute RSV infection. We initially evaluated IFN-γ levels, as it is a common pro-inflammatory cytokine produced by CD8 T cells following viral infection. IFN-γ protein levels were significantly (*p*<0.001) increased in both the lung and serum at day 2 p.i. of M2_82_-immunized mice as compared to the control group (Fig 4A and 4B). IFN-γ levels in M2_82_-immunized mice remained elevated above controls at day 4 p.i., but the overall amount was reduced as compared to day 2 following infection. Interestingly, challenge with IAV-M2_82_ resulted in significantly (*p*<0.001) greater IFN-γ protein levels in the lung at day 2 and day 4 p.i. as compared to challenge with RSV (S13A Fig). In contrast, serum IFN-γ and lung TNF levels in IAV-M2_82_-infected mice were reduced (*p*<0.001) at day 2 but increased (*p*<0.001) at day 4 p.i. when compared to the levels observed following RSV challenge (S13B and S13C Fig).

To determine the in vivo source of IFN-γ in RSV-infected immunized mice, we treated mice with brefeldin A (BFA) to capture cells producing IFN-γ via intracellular staining and flow cytometry [43]. Leukocytes producing IFN-γ in vivo were readily identified using this previously established method (S14A Fig). Upon evaluation of the primary leukocyte populations present in the lung following RSV infection, only lymphocytes had produced IFN-γ in immunized mice at day 2 p.i. (Fig 4C). Only a small frequency of CD4 T cells and NK cells secreting IFN-γ were observed at day 2 p.i., whereas approximately 45% of CD8 T cells were producing IFN-γ (Fig 4C). These data also correlate with the rapid and transient increase in the amount of IFN-γ protein we observed in the lung and serum at day 2 p.i., as virtually no IFN-γ-producing cells were recovered on day 5 p.i. (S14B Fig). The IFN-γ secreting CD8 T cells were largely CD11a^hi^, indicating that the majority were antigen-experienced T cells (Fig 4D) [44]. When comparing M2_82_-specific T lymphocytes to all other CD8 T cells in the lung at day 2 p.i., half of M2_82_-specific CD8 T cells produced IFN-γ, whereas almost 30% of the remaining CD8 T cells were also producing IFN-γ (Fig 4E). These results indicate that both M2_82_-specific and bystander antigen-experienced CD8 T cells secrete IFN-γ in immunized mice early following RSV infection. Lastly, we assessed IFN-γ production by CD8 T cells in the respiratory tract (lung and BAL), mediastinal lymph node (mLN), and periphery (spleen and PBL). The majority of CD8 T cells secreting IFN-γ were localized to the lung and BAL (Fig 4F). In contrast, a relatively low frequency of CD8 T cells produced IFN-γ in the spleen and PBL, and virtually no IFN-γ production was observed in the mLN (Fig 4F). Taken together, these data demonstrate that antigen-experienced CD8 T cells in the airways of immunized mice secrete IFN-γ early following RSV infection leading to increased IFN-γ levels in both the lung and the periphery.

### Neutralization of IFN-γ in the airways ameliorates enhanced disease and prevents mortality

**Fig 5:**
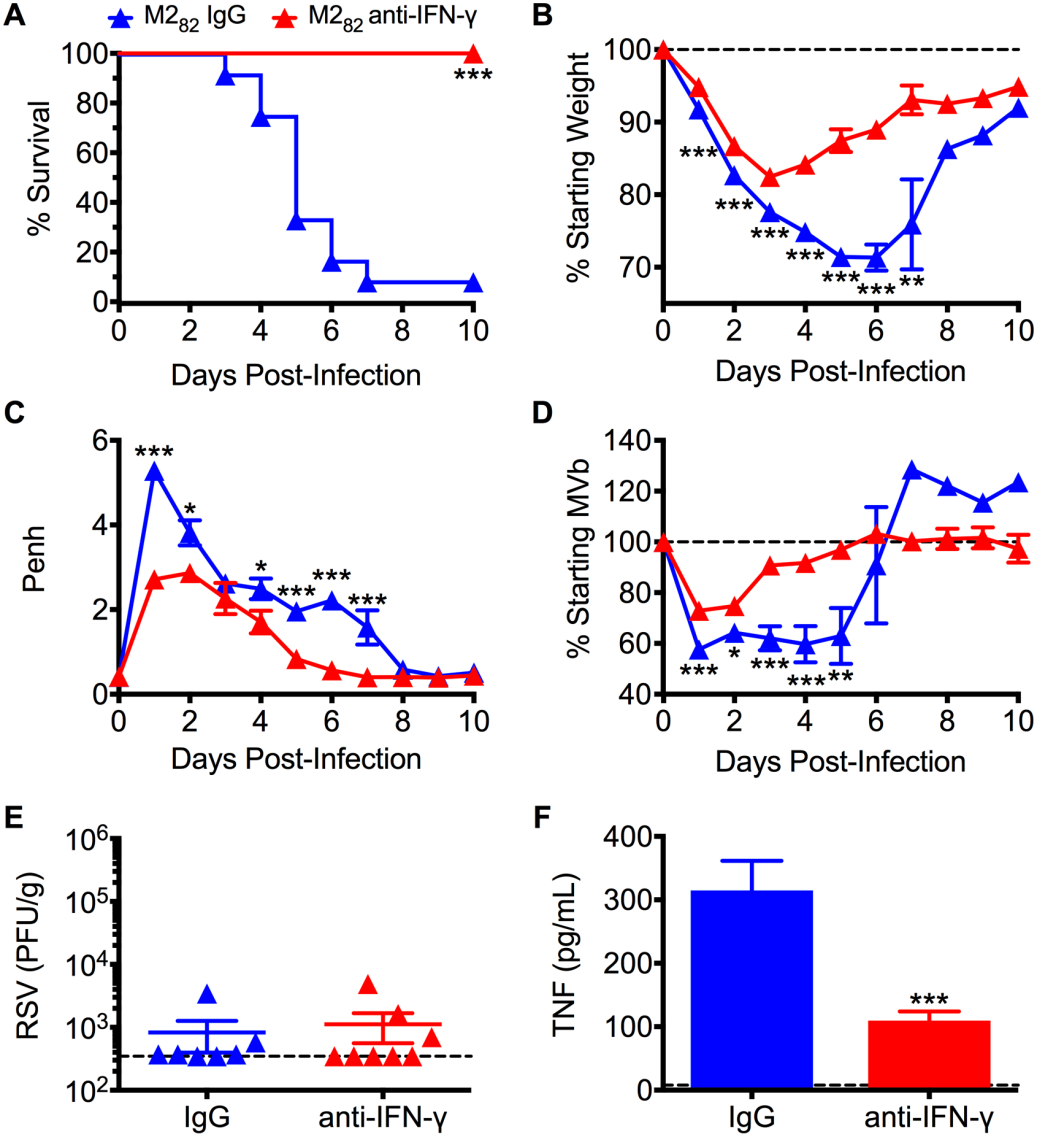
Neutralization of airway IFN-γ prevents lethal immunopathology associated with pre-existing CD8 T cell memory. M2_82_-immunized mice were treated with either control IgG or anti-IFN-γ antibody i.n. once during the time of RSV challenge. DC-LM-immunized mice were assessed daily for (A) survival, (B) weight loss, (C) Penh, and (D) MVb following infection. (E) RSV lung titers were determined at day 4 p.i. (F) TNF protein levels in the lung were quantified at day 2 p.i. Data are represented as mean ± SEM of two independent experiments (*n* = 11 for (A-D) and *n* = 8 for E-F)). Statistical analysis was performed using Student’s *t* test, * *p*<0.05, ** *p*<0.01, *** *p*<0.001.

Due to the increased systemic IFN-γ levels largely produced by antigen-experienced CD8 T cells in the respiratory tract, we next determined if IFN-γ was necessary to mediate the severe immunopathology in immunized mice. To evaluate the role of IFN-γ, we treated M2_82_-immunized mice with either control IgG or anti-IFN-γ neutralizing antibody administered i.n at the time of RSV challenge. While high mortality was observed in the IgG-treated group, neutralization of IFN-γ led to the survival of all immunized mice (Fig 5A). In addition, weight loss and respiratory dysfunction were significantly (*p*<0.05) reduced in immunized mice following IFN-γ neutralization as compared to the IgG-treated mice (Fig 5B–5D). Neutralization of IFN-γ did not significantly impact virus titers in the lung at day 4 p.i., suggesting that IFN-γ does not contribute to pathogen clearance in this prime-boost immunization model (Fig 5E). Neutralization of IFN-γ resulted in significantly (p<0.001) decreased TNF levels in the lung at day 2 p.i. compared to IgG-treated controls (Fig 5F). Overall, our results suggest that antigen-experienced CD8 T cells rapidly secrete IFN-γ, which mediates lethal immunopathology following RSV infection in DC-LM-immunized mice by promoting the production of TNF by other cell populations.

## Discussion

Current RSV vaccine development and assessment is focused upon induction of a strong humoral immune response [45]. In contrast, the capacity of cellular immunity to provide protection against an RSV infection has received less attention. Here we evaluated the capacity of memory CD8 T lymphocytes to protect against an RSV infection. Our results demonstrate that memory CD8 T cells, in the absence of RSV-specific CD4 T cell memory or antibodies, promote enhanced viral clearance following RSV challenge. However, pre-existing RSV-specific memory CD8 T cells also mediate exacerbated disease severity and lethal immunopathology. The CD8 T cell response has been previously shown to contribute to weight loss and illness following an acute RSV infection [12]. Therefore, the CD8 T cell response plays a crucial role in both viral clearance and immunopathology during primary and secondary responses.

A number of previous reports have shown that the adoptive transfer of activated effector RSV-specific CD8 T cells, in vitro stimulated T cell lines, or in vitro propagated T cell clones results in enhanced RSV clearance from the lung following RSV challenge. These effector CD8 T cell transfers were also associated with increased weight loss, indicating that infusion of effector CD8 T cells leads to the induction of increased systemic disease [20–23]. Thus, our studies contrast substantially with these previous studies as we have examined the protective capacity of in vivo generated RSV-specific memory CD8 T cells that have not undergone in vitro activation or restimulation.

Vallbracht et al. reported that mutation of the M2_82_ epitope sequence within the RSV genome results in reduced T cell-mediated immunopathology following an acute infection [32]. However, the enhanced disease severity associated with pre-existing RSV-specific memory CD8 T cells we observe here was not limited to either a specific epitope or RSV protein. DC-LM immunization targeting either the M2_82_ or F_85_ epitope resulted in exacerbated disease and high mortality following RSV challenge. Furthermore, immunization against the immunodominant M_187_ epitope in C57BL/6 mice also caused increased weight loss and pulmonary dysfunction, but no mortality following RSV infection. The lack of mortality in the immunized C57BL/6 mice may be due to M_187_-specific CD8 T lymphocytes having superior cytolytic function with limited immunopathology as compared to M2_82_-specific CD8 T cells [46].

Interestingly, the lethal immunopathology associated with pre-existing memory CD8 T cells was unique to the context of an RSV infection. Memory CD8 T cells are protective and do not exacerbate disease severity with other respiratory viral infections such as IAV or SARS-CoV [25, 26]. Consistent with memory CD8 T cells being able to provide protection against IAV infection [25], we show that RSV M2_82_-specific memory CD8 T cells mediate protection following a lethal IAV-M2_82_ infection. A systemic increase of IFN-γ was seen in immunized mice early 2 days following RSV infection. Antigen-experienced CD8 T cells in the airways were the primary source of IFN-γ. The presence of IFN-γ-producing CD8 T cells only in the airways suggests that production was in response to antigen stimulation given the strong tropism of RSV to the epithelium and alveolar cells of the respiratory tract [47–49]. Interestingly, peak IFN-γ production occurred prior to the significant accumulation and/or expansion of virus-specific CD8 T cells within the lung by day 5 p.i. This observation is similar to work by Liu et al. showing IFN-γ production by CD8 T cells prior to expansion of the CD8 T cell response following lymphocytic choriomeningitis virus (LCMV) infection [43]. Neutralization of IFN-γ prevented all mortality and reduced disease severity in immunized mice without impacting RSV titers. These data indicate that IFN-γ promotes the severe immunopathology, but does not contribute to viral clearance.

A robust IFN-γ response may explain why memory CD8 T cells protect against a lethal IAV-M2_82_ and not an RSV infection. Antibody-mediated neutralization of IFN-γ does not impact RSV viral titers during an acute infection indicating a minor role in viral clearance [42]. Induction of a Th1-biased immune response is important to prevent the pathology associated with a Th2-skewed response [42, 50–52]. Nonetheless, IFN-γ still contributes to minimal immunopathology associated with an acute RSV infection [42, 52]. However, treatment of mice with recombinant IFN-γ at early timepoints has been shown to protect against lethal IAV infection with no difference in viral clearance [53]. This result is consistent with our study showing that DC-LM immunized mice receiving a lethal IAV infection have significantly increased IFN-γ protein levels in the lung at early time points as compared to RSV challenged mice. Together, these results suggest that supplemental IFN-γ limits the severe pathology associated with a lethal IAV infection without contributing to viral clearance. Therefore, IFN-γ can play distinct roles by either contributing to immunopathology or ameliorating disease dependent upon the respiratory virus infection.

Other CD8 T cell functions were not required to mediate the exacerbated disease observed in DC-LM-immunized mice. Immunized mice deficient in perforin exhibited similar disease severity as WT controls following RSV challenge. In addition, all perforin-deficient mice eventually succumbed to infection whereas typically 10–20% of WT immunized mice survive. It has been previously demonstrated that IFN-γ and TNF levels in perforin-deficient mice are elevated following an acute RSV infection, which likely contributes to the accelerated mortality we observed in M2_82_-immunized perforin-deficient mice [42]. A role for regulating CD8 T cell expansion and cytokine production by perforin to prevent mortality has also been demonstrated for secondary CD8 T cell responses against LCMV [54, 55]. Lastly, the neutralization of TNF also improved survival and ameliorated disease in immunized mice following RSV infection. The cytokine TNF is known to contribute to both weight loss and inflammation during acute RSV infection [42]. Therefore, TNF plays a critical role in mediating immunopathology during both primary and secondary RSV infections.

Our results are in contrast to work by Lee et al. showing enhanced RSV clearance with reduced disease severity mediated by vaccine-elicited memory CD8 T cells [56]. The DC-LM immunization utilized in our studies resulted in dramatically increased numbers of RSV-specific memory T cells compared to the immunization strategy employed by Lee et al. [56]. This disparity in RSV-specific memory CD8 T cell numbers prior to RSV challenge may account for the observed difference in outcome between our studies. It remains controversial whether a strong CD8 T cell response is desirable during RSV infection in humans. Experimental RSV infection of adult humans revealed that greater frequencies of virus-specific CD8 T cells in the BAL correlated with reduced clinical disease symptoms [57]. In contrast, a greater ratio of CD8 to CD4 T cells in the airways is associated with acute lung injury during common respiratory tract infections such as RSV [58].

RSV-specific CD8 T cell responses have also been examined in humans following RSV infection. Consistent with their critical role in clearing an acute infection, a study by Weilliver et al. found that children with a fatal primary RSV infection had fewer CD8 T cells in their lung tissue than normal controls suggesting that patients with severe lower respiratory tract illness may have an insufficient cell-mediated immune response [59]. In contrast, a report by Heidema et al. examining CD8 T cells in the airways of infants with a severe primary RSV infection was able to readily identify RSV-specific CD8 T cells in the airways with no significant difference in the number recovered between infants with more versus less severe disease [60]. Thus, it is currently unclear if CD8 T cells contribute to immunopathology during an acute RSV infection in infants.

The role of RSV-specific memory CD8 T cells in humans has been difficult to address due to the difficulty in obtaining CD8 T cells from the airways. A recent report by Jozwik et al. using the human RSV challenge model has sought to address this issue. In adult volunteers experimentally infected with RSV, Jozwik et al. found that a higher baseline frequency of RSV-specific CD8 T cells in the airways correlated with a lower cumulative symptom score following RSV challenge [57]. Our results obtained by administration of a local IAV-M2_82_ boost are consistent with this notion. Our data suggest that systemic RSV-specific memory CD8 T cells are more prone to causing immunopathology, possibly in part due to their delay in reaching the lungs. However, the use of the human RSV challenge model cannot evaluate the protective capacity of memory CD8 T cells in the absence of RSV-specific memory CD4 T cells and antibodies as we have done here using our animal model. Thus, we believe our results are applicable to humans in cases where vaccination of an RSV seronegative individual would primarily elicit a CD8 T cell response.

Our study defines a clear role for memory CD8 T cells following RSV infection. Pre-existing CD8 T cell memory contributes to enhanced viral clearance upon RSV challenge, but also mediates severe immunopathology in contrast to many other viral infections. The outcome is compelling given the high rate of mortality, which is unusual in this RSV infection model. These data highlight how complex and unique the RSV-induced immune response is in contrast to other respiratory viral infections. Our results indicate that epitope-based cellular vaccines against RSV may have detrimental consequences. Our data also support that caution must be exercised during evaluation of any RSV vaccine candidate, particularly when robust memory T cell responses are involved in order to prevent the induction of immunopathology.

## Materials and methods

### Mice

Female BALB/cAnNCr mice between 6–8 wk old were purchased from the National Cancer Institute (Frederick, MD). Female H-2^d^ perforin-deficient mice were provided by Dr. John Harty (University of Iowa, Iowa City, IA) [61, 62].

### Ethics statement

All experimental procedures utilizing mice were approved by the University of Iowa Animal Care and Use Committee under Animal Protocols #4101196 and #7041999. The experiments were performed under strict accordance to the Office of Laboratory Animal Welfare guidelines and the PHS Policy on Humane Care and Use of Laboratory Animals.

### Prime-boost immunization

Memory CD8 T cells were induced using a DC-LM, prime-boost immunization regimen. BALB/c mice were injected intraperitoneally (i.p.) with 5 x 10^6^ B16 melanoma cells that express fms-related tyrosine kinase 3 ligand (B16-FLT3L). After 14 days, mice were injected i.v. with 1–2 μg lipopolysaccharide (LPS) to mature DCs. 24 hrs later, spleens were harvested and digested in HBSS containing 60 U/mL DNase I (Sigma-Aldrich) and 125 U/mL collagenase (Invitrogen) while gently shaking for 20 mins at 37°C. Spleens were made into single-cell suspensions and incubated with a 2 μM concentration of either M2_82-90_ or F_85-93_ peptide for 2 hrs at 37°C while rocking. DCs were isolated using anti-CD11c microbeads (Miltenyi Biotec) and sorted via positive selection on an autoMACS separator (Miltenyi Biotec). Mice were primed with 5 x 10^5^ peptide-pulsed DCs. DC-immunized mice were boosted with 5 x 10^6^ *actA*-deficient LM that express either M2_82_ or F_85_ administered i.v. 7 days later. 28–42 days following the LM boost, mice were infected with either RSV or IAV-M2_82_. Control mice were primed with DCs incubated without peptide and boosted with an *actA*-deficient LM that does not express any RSV-derived epitopes [63]. The recombinant LM strains were created using pPL2 integration vector [64]. Target DNA was inserted at digested BamH1 and PstI sites and ligated in *Escherichia coli* XL1-Blue cells. Recombinant chloramphenicol-resistant plasmids were conjugated in *E*. *coli* SM10 cells along with the 10403S strain of LM that is resistant to streptomycin [65] on brain heart infusion agar plates. Growth from previous step were streaked out on selective brain heart infusion agar plates to select chloramphenicol- and streptomycin-resistant colonies that contain pPL2 integrated into the 10403S LM strain. Recombinant LM were grown in tryptic soy broth (35.6g/L) containing 50 mg/mL streptomycin.

### Viruses and infection

The A2 strain of RSV was a gift from Dr. Barney Graham (National Institutes of Health, Bethesda, MD). The A2-line19F strain was a gift from Dr. Martin Moore (Emory University, Atlanta, GA). RSV strains were propagated in HEp-2 cells (ATCC). Mice were infected i.n. with 1.0–1.7 x 10^6^ PFU of purified RSV. For RSV purification, 50% polyethylene glycol was added to crude RSV for a final dilution of 1:5. The RSV preparation was mixed at 4°C for 2 hrs and centrifuged at 7300 g for 30 mins in a swing bucket rotor. Pellets were resuspended in 20% sucrose solution and placed on top of 60% and 35% sucrose layers and centrifuged at 170,000 g for 1 hr. Purified RSV at the interface between the 35% and 60% sucrose layers was collected and stored at -80°C. All solutions were created in a buffer containing 0.15 M NaCl, 0.05M Tris-HCl, and 0.001M EDTA. For mock infections, mice were administered an equivalent volume of sterile PBS. Recombinant IAV-M2_82_ was kindly provided by Dr. Ryan Langlois (University of Minnesota, Minneapolis, MN). The virus was created using standard reverse genetics as previously described [66], rescued, and grown in 10 day-old embryonated chicken eggs (Charles River). M2_82_ epitope was inserted into the mRNA nucleotide position 186 encoding the neuraminidase stalk region, which is known to tolerate such insertions [67]. For lethal heterologous IAV infections, mice were challenged i.n. with a 5 LD_50_ dose representing 1 x 10^3^ PFU of recombinant IAV-M2_82_ virus. For sublethal IAV infections, mice were infected i.n. with a 0.1 LD_50_ dose representing 20 PFU of IAV-M2_82_. In certain experiments, mice were boosted with IAV-M2_82_ and given a 0.1 LD_50_ dose i.n. 7 days following the DC-M2_82_ prime i.v.

### Plaque assay for RSV and IAV

Whole lungs were harvested from mice, weighed, mechanically homogenized, and supernatant was stored at -80°C until further use. 1:10 serial dilutions of supernatants were performed and incubated on Vero cells (ATCC) in 6-well plates for 90 mins at 37°C. Plates were overlaid with a 1:1 mixture of 2X Eagle minimum essential medium (2X EMEM, Lonza, Walkersville, MD) and 1% SeaKem ME agarose (Cambrex, North Brunswick, NJ). Following 5 days of incubation at 37°C, 5% CO_2_, plates were stained with a 1:1 mixture of 2X EMEM and 1% agarose containing 0.015% neutral red (Sigma-Aldrich). Plaques were counted after 24–48 hrs. For determination of IAV titers, lungs were processed in the same manner as for RSV plaque assay. MDCK (ATCC) cells in 6-well plates were washed 3 times with room temperature sterile PBS adding 1 mL of sterile Dulbecco’s modified Eagle’s medium afterwards. Plates were infected with 100 μl of serially diluted IAV-infected lung samples (10-fold dilutions) for 1 hr at 37°C. Plates were washed twice with sterile room temperature PBS. Wells were overlaid with 2 mL of a 1:1 mixture of 2X EMEM and 1.6% agarose containing 1 mg/mL TPCK-trypsin and incubated at 37°C, 5% CO_2_ for 3 days. Agarose plugs were carefully removed, and monolayers were fixed with 2 mL 70% ethanol for 20 mins at room temperature. Monolayers were stained with 1 mL of 1% crystal violet in methanol for 10 mins at room temperature, and plates were washed in a pool of warm water. Plates were allowed to dry overnight, and plaques were counted the next morning.

### Assessment of pulmonary function and weight loss

Pulmonary function of mice was evaluated using unrestrained whole-body plethysmography. Enhanced pause (Penh) and respiratory minute volume (MVb) were measured using a whole-body plethysmograph (Buxco Electronics, Wilmington, NC) and averaged over a 5 min period. Weight loss was tracked daily following RSV or IAV infection of mice. Mice that were at or below 70% of their starting weight were euthanized.

### In vivo antibody-mediated depletion or neutralization

For IFN-γ and TNF neutralization, mice were treated i.n. with 200 μg of anti-IFN-γ (clone XMG1.2) or anti-TNF (clone MP6-XT22) antibody during RSV challenge. For controls, mice were administered a matching dose of control isotype IgG antibody.

### Histology

Whole lungs were harvested on day 5 following RSV challenge and fixed in 10% neutral buffered formalin (Fisher Scientific). Lungs were processed as previously described [68] and stained with H&E for routine evaluation. Representative images of lung sections were taken at 20X, 200X, and 400X magnification for each immunization regimen. Tissues were examined and scored in a manner masked to experiment groups [69]. Each sample was assessed for evidence of DAD. Histopathologically, early stages of DAD include alveolar septal injury, such as cellular sloughing, necrosis, hyaline membrane formation, hemorrhage, and early cellular infiltrates. DAD scores were assigned as follows: 1—absence of cellular sloughing and necrosis; 2—Uncommon solitary cell sloughing and necrosis; 3—Multifocal cellular sloughing and necrosis with uncommon septal wall hyalinization; 4—Multifocal cellular sloughing and necrosis with common and prominent hyaline membranes.

### Cytokine multiplex and ELISA

Serum was collected and whole lungs were harvested on days 0, 2, and 4 p.i. Lungs were disrupted using a tissue homogenizer (Ultra-Turrax T25; IKA Works, Inc., Wilmington, NC) in Cell Lysis Buffer (eBioscience). Lung homogenates were centrifuged at 2000 rpm for 10 mins, and supernatants were collected. The protein levels of 20 different cytokines and chemokines in the lung and serum were determined using a ProcartaPlex Multiplex Immunoassay kit (eBioscience) according to the manufacturer’s instructions. The assay was run on a BioPlex instrument (Bio-Rad, Hercules, CA). Lung and serum IFN-γ levels were determined by ELISA as previously described (eBioscience) [68]. Lung TNF levels were determined using a mouse TNF ELISA kit (Invitrogen) according to manufacturer’s instructions.

### Flow cytometry analysis and tissue collection

Lung and BAL were harvested from mice as previously described [70, 71]. Spleens and mLN were gently dissociated between the frosted ends of microscope slides. Cells from the lung, BAL, spleen, mLN, and PBL were stained for extracellular surface molecules with antibodies specific to CD11c (clone N418), Siglec F (BD Biosciences, clone E50-2440), F4/80 (clone BM8), Ly6c (clone HK1.4), Ly6g (clone 1A8), CD49b (clone DX5), NKp46 (clone 29A1.4), CD11a (clone M17/4), CD90.2 (clone 53–2.1), CD3ε (clone 145-2C11), CD4 (clone GK1.5), CD8 (clone 53–6.7), CD69 (clone H1.2F3), and CD103 (clone 2E7) for 30 mins at 4°C and fixed with fix/lyse solution (eBioscience) for 10 mins at room temperature. After extracellular staining, cells were stained for FoxP3 (eBioscience clone FJK-16s) with transcription factor staining buffer set (eBioscience) according to manufacturer’s instructions. For intracellular cytokine staining, cells were stimulated for 5 hrs at 37°C with 2 μM M2_82-90_ peptide in 10% FCS-supplemented RPMI. Stimulated cells were stained for surface markers as indicated above and then stained intracellularly with antibodies specific to IFN-γ (clone XMG1.2) and TNF (clone MP6-XT22) in FACS buffer containing 0.5% saponin (Sigma-Aldrich) for 30 mins at 4°C. Total numbers of cytokine producing cells were calculated after subtraction of background staining from BFA-only controls. All monoclonal antibodies were purchased from BioLegend unless otherwise stated. Stained cells were run on LSRFortessa and analyzed with FlowJo (Tree Star, Ashland, OR) software. Cell types were phenotyped as follows: CD8 T cells (CD90.2^+^CD8^+^), CD4 T cells (CD90.2^+^CD4^+^), Tregs (CD90.2^+^CD4^+^FoxP3^+^), NK cells (CD3ε^-^CD49b^+^NKp46^+^), monocytes (CD11c^+^F4/80^+^), eosinophils (SiglecF^+^CD11c^lo^), and neutrophils (Siglec F^-^CD11c^-^Ly6c^+^Ly6g^+^).

### Intravasuclar staining

Mice were injected i.v. with 1 μg CD45-FITC (CD45 labeled with fluorescein isothiocyanate) (clone 30-F11) antibody 3 mins prior to euthanasia. Cells from the lung were processed as previously described [37].

### In vivo assessment of IFN-γ-producing cells

Analysis of IFN-γ-producing cells was performed using in vivo BFA Administration [43]. Mice were injected i.v. with 250 μg BFA (0.5 mg/mL; Sigma) in 500 μl PBS, and lungs, BAL, spleen, mLN, and PBL were harvested 6 hrs later. Leukocytes were stained as indicated above.

### Statistical analysis

All statistical analyses are described in each figure legend and were performed using Prism software (GraphPad Software, San Diego, CA). Data were evaluated using unpaired, two-tailed Student’s *t* tests between two groups or one-way ANOVA with Tukey-Kramer post-test analyses for more than two groups to determine if there was a statistical significance of at least α = 0.05. Asterisks or pound signs are used to define a difference of statistical significance between the indicated group and its respective control group unless otherwise indicated by a line or stated in the figure legend.

## Supporting information

S1 Fig Specific cell types are altered by robust memory CD8 T cell responses following RSV infection. Total numbers of CD4 T cells, Tregs, monocytes, eosinophils, neutrophils, and NK cells were determined on days 0, 4, and 5 following RSV infection of immunized mice. Data are represented as mean ± SEM of two independent experiments (*n* = 8 mice). Groups within each cell type were compared using one-way ANOVA, * *p*<0.05, ** *p*<0.01, *** *p*<0.001.
(PDF)

S2 Fig Increased levels of cell death in the airways of immunized mice. Lungs from naive, control, and M2_82_-immunized mice were collected at day 5 following RSV infection and processed for H&E staining. Representative photos of lung sections were captured at 20X magnification. Arrows point to regions of cell death and debris accumulation in the airways, which show up as pink areas.
(PDF)

S3 Fig Diffuse alveolar damage is present in the lungs of immunized mice. Lungs from M2_82_-immunized mice were collected at day 5 following RSV infection and processed for H&E staining. Representative photos of lungs sections showing a range of lesions consistent with early diffuse alveolar damage (DAD) were captured at 400X magnification. Hyaline membranes are indicated by the black arrows. (A) Scattered cellular sloughing, necrotic debris (inset), and increases in cellularity by immune cell infiltration were present. (B) Regions of alveolar hemorrhage adjacent to hyaline membranes are indicated by asterisks. (C) The formation of prominent hyaline membranes in multiple regions of the lung were visible.
(PDF)

S4 Fig F_85_-specific memory CD8 T cells also mediate enhanced RSV clearance and immunopathology. Naive BALB/c mice were control- or F_85_-immunized as in Fig 1 and infected with RSV 42 days later. (A) F_85_-tetramer^+^ CD8 T cell response was measured in the PBL following LM booster immunization. (B) RSV titers at day 4 p.i. were determined via plaque assay in the lung. (C) Survival, (D) weight loss, (E) Penh, and (F) MVb were evaluated daily following RSV infection. Results are presented as mean ± SEM of two independent experiments (*n* = 8 mice). Groups were compared using Student’s *t* test, * *p*<0.05, ** *p*<0.01, *** *p*<0.001.
(PDF)

S5 Fig Pre-existing CD8 T cell memory in C57BL/6 mice promotes enhanced disease severity without mortality. Naive C57BL/6 mice were immunized as in Fig 1, but targeting the immunodominant CD8 epitope M_187_. (A) Frequency of M_187_-specific CD8 T cells was determined by tetramer staining the PBL following the LM booster immunization. (B) RSV titers in the lung were assessed at day 4 following challenge. (C) Penh, (D) MVb, and (E) weight loss were monitored daily following RSV infection. Data are presented as mean ± SEM of two independent experiments (*n* = 10 mice for viral titers; *n* = 8 for disease assessment). Groups were compared using Student’s *t* test, * *p*<0.05, ** *p*<0.01,*** *p*<0.001.
(PDF)

S6 Fig CD8 T cell-mediated immunopathology in immunized mice is RSV strain-independent. M2_82_-immunized mice were challenged with either A2 or A2-line19F RSV strains and monitored daily for (A) survival, (B) weight loss, (C) Penh, and (D) MVb. Data are represented as mean ± SEM of two independent experiments (*n* = 10 mice).
(PDF)

S7 Fig Cellular accumulation in the lung of prime-boosted mice is similar following challenge with either RSV or IAV-M2_82_. Control and M2_82_-immunized mice were challenged with a 5 LD_50_ dose of IAV-M2_82_. (A) Total CD8 and (B) M2_82_-specific CD8 T cells in the lungs of immunized mice at days 0, 4, and 5 p.i. (C) Total numbers of CD4 T cells, Tregs, monocytes, eosinophils, neutrophils, and NK cells in the lungs on days 0, 4, and 5 p.i. Data are represented as mean ± SEM of two independent experiments (*n* = 8 mice). Groups within each cell type were compared using one-way ANOVA, * *p*<0.05, ** *p*<0.01, *** *p*<0.001.
(PDF)

S8 Fig Memory CD8 T cells do not enhance disease severity following sublethal IAV-M2_82_ infection. Control- and M2_82_-immunized mice were challenged with a sublethal 0.1 LD_50_ dose of IAV-M2_82_ and assessed daily for (A) Penh, (B) MVb, and (C) weight loss. Data are presented as mean ± SEM of two independent experiments (*n* = 8 mice for control group and *n* = 10 for M2_82_ group). Groups were compared using Student’s *t* test, * *p*<0.05, ** *p*<0.01, *** *p*<0.001.
(PDF)

S9 Fig Local immunization induces M2_82_-specific resident memory CD8 T cells within the lung tissue. DC-M2_82_-primed mice were either not boosted or boosted with either LM-M2_82_ i.v. or IAV-M2_82_ i.n. 33 days post-boost mice were administered anti-CD45 antibody i.v. 3 mins prior to harvest to stain cells within the vasculature. Representative flow plots (A) and total numbers (B) of M2_82_-tetramer^+^ CD8 T cells in the lung. Representative flow plots (C) and total numbers (D) of IV stain^-^ M2_82_-tetramer^+^ CD8 T cells within the lung tissue. Representative flow plots (E) and total numbers (F) of resident memory IV stain^-^ M2_82_-specific CD8 T cells in the lung. Data are represented as mean ± SEM of two independent experiments (*n* = 8 mice). Groups were compared using one-way ANOVA, *** *p*<0.001.
(PDF)

S10 Fig Resident memory CD8 T cells induced by local immunization prevent fatal immunopathology following RSV challenge. DC-M2_82_-primed mice were either not boosted or boosted with either LM-M2_82_ i.v. or IAV-M2_82_ i.n. Mice were assessed for (A) survival, (B) weight loss, (C) Penh, and (D) MVb following RSV infection. Data are represented as mean ± SEM of two independent experiments (*n* = 10 mice). Groups were compared using one-way ANOVA, * *p*<0.05, ** *p*<0.01, *** *p*<0.001. Asterisks represent statistical significance between LM-M2_82_ and IAV-M2_82_ groups.
(PDF)

S11 Fig Perforin is not necessary to mediate enhanced disease in immunized mice. M2_82_ DC-LM-immunized WT and perforin knock out (KO) mice were challenged with RSV 28 days later and monitored daily for (A) survival, (B) weight loss, (C) Penh, and (D) MVb. Data are presented as mean ± SEM of two independent experiments (*n* = 11 WT; *n* = 14 perforin KO).
(PDF)

S12 Fig TNF is necessary for lethal immunopathology associated with robust memory CD8 T cell responses. M2_82_-immunized mice were treated with 200 μg of either IgG or anti-TNF antibody i.n. during the time of RSV infection. (A) Survival, (B) weight loss, (C) Penh, and (D) MVb were assessed daily following RSV challenge. (E) RSV titers in the lung were determined via plaque assay at day 4 p.i. (F) TNF protein amounts were quantified at days 0, 2, and 4 p.i. in the lung and serum of control- and M2_82_-immunized mice. Data are presented as mean ± SEM of two independent experiments (*n* = 11 in (A-D); *n* = 8 in (E); *n* = 6 for control and *n* = 8 for M2_82_ in (F)). Statistical comparisons were performed using Student’s *t* test, * *p*<0.05, ** *p*<0.01, *** *p*<0.001.
(PDF)

S13 Fig IAV-M2_82_ induces greater levels of IFN-γ protein in the lungs of immunized mice than RSV. Control and M2_82_-immunized mice were challenged with either RSV or a 5 LD_50_ dose of IAV-M2_82_. IFN-γ protein levels were determined in the (A) lung and (B) serum at 0, 2, and 4 days p.i. by ELISA. (C) TNF protein levels were quantified in the lung at 0, 2, and 4 days p.i. by ELISA. Data are represented as mean ± SEM of two independent experiments (*n* = 8 mice). Groups were compared using one-way ANOVA, * *p*<0.05, ** *p*<0.01, *** *p*<0.001.
(PDF)

S14 Fig IFN-γ production by CD8 T cells is early following RSV infection in immunized mice. DC-LM-immunized mice were administered 250 μg BFA i.v. 6 hours prior to organ collection. (A) Representative flow plots of IFN-γ production gated on CD4 and CD8 T cells at day 2 p.i. in the lung. (B) IFN-γ secretion by leukocyte populations in the lung at day 5 following either mock or RSV challenge. Data are presented as mean ± SEM of two independent experiments (*n* = 8 mice).
(PDF)
