# Supplementary figures and images for: Memory CD8 T cells mediate severe immunopathology following respiratory syncytial virus infection
Source: PLoS Pathog. 2018 Jan 2;14(1):e1006810. doi: 10.1371/journal.ppat.1006810 (PMC5766251; doi:10.1371/journal.ppat.1006810)

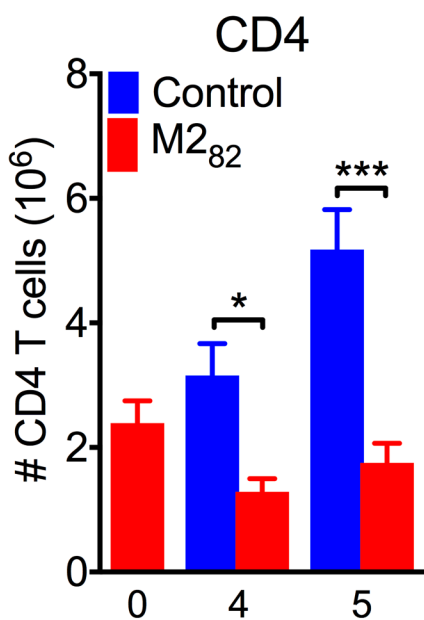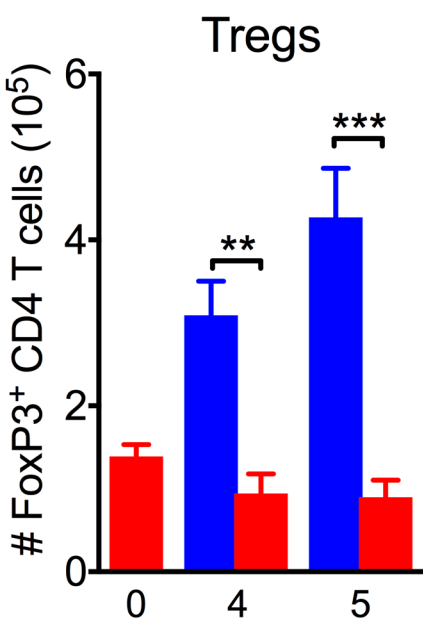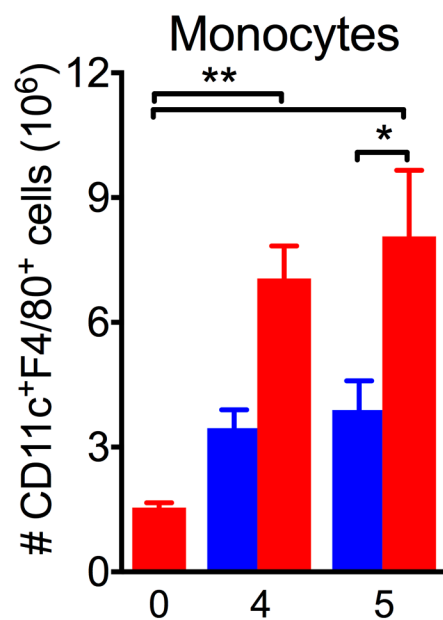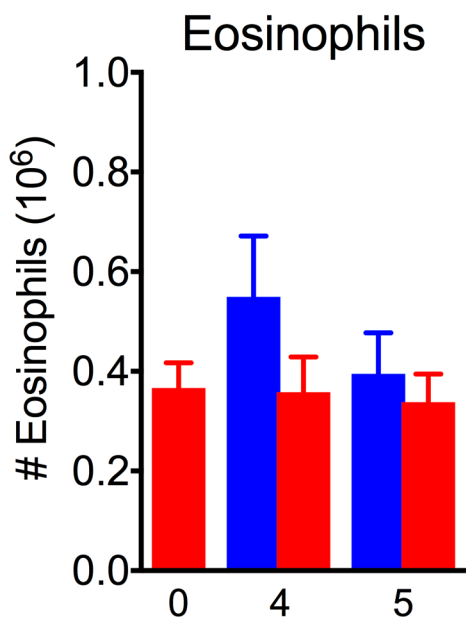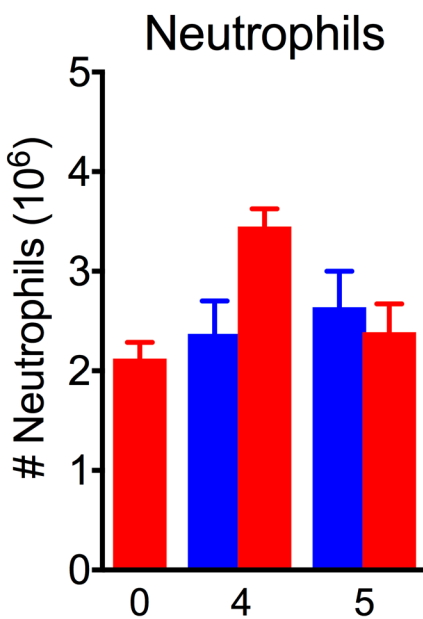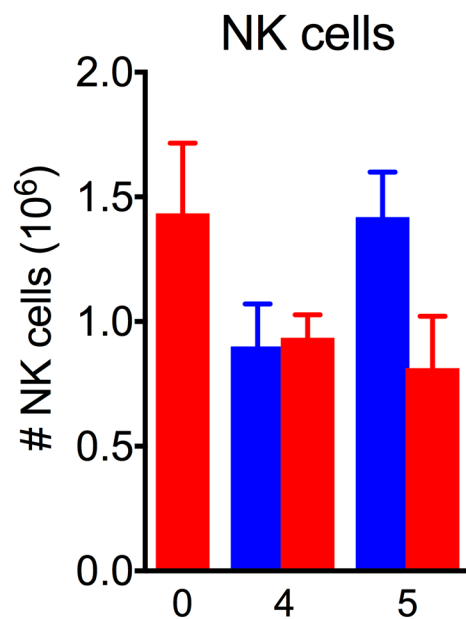

Supplement: S1 Fig — Total numbers of CD4 T cells, Tregs, monocytes, eosinophils, neutrophils, and NK cells were determined on days 0, 4, and 5 following RSV infection of immunized mice. Data are represented as mean ± SEM of two independent experiments (n = 8 mice). Groups within each cell type were compared using one-way ANOVA, * p<0.05, ** p<0.01, *** p<0.001. (PDF) [file ppat.1006810.s001.pdf]

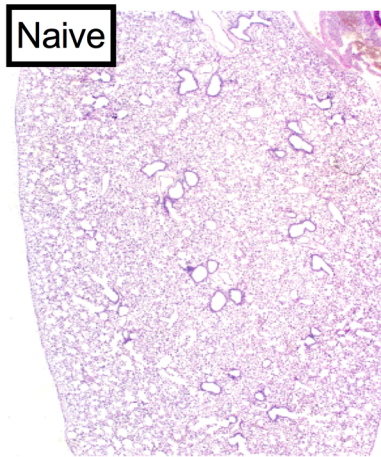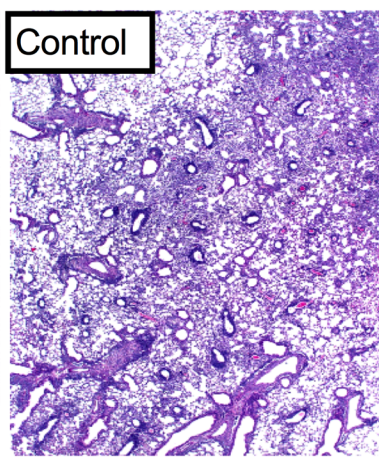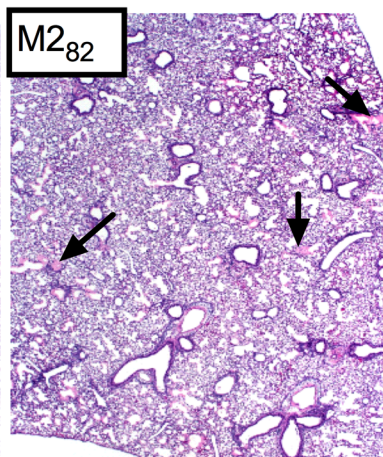

Supplement: S2 Fig — Lungs from naive, control, and M282-immunized mice were collected at day 5 following RSV infection and processed for H&E staining. Representative photos of lung sections were captured at 20X magnification. Arrows point to regions of cell death and debris accumulation in the airways, which show up as pink areas. (PDF) [file ppat.1006810.s002.pdf]

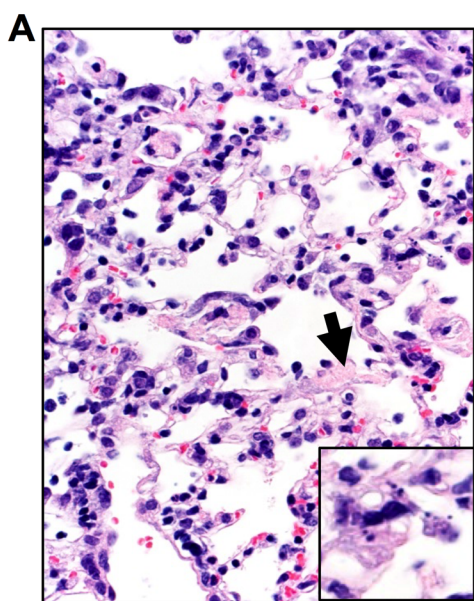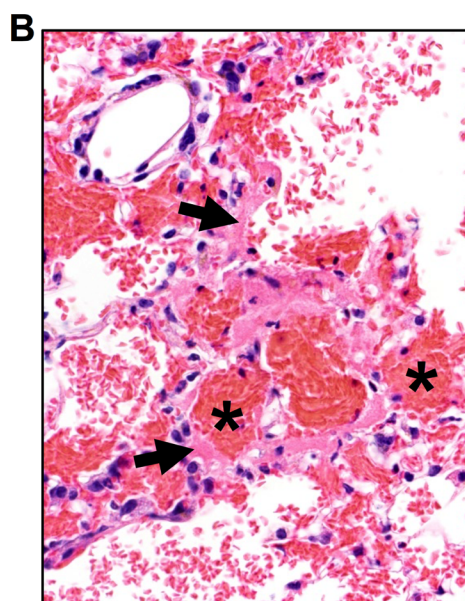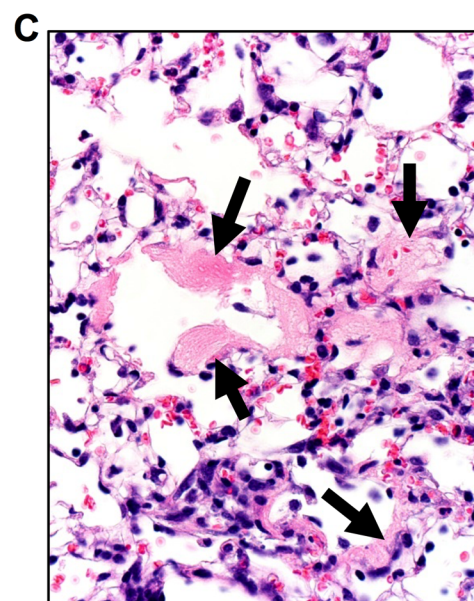

Supplement: S3 Fig — Lungs from M282-immunized mice were collected at day 5 following RSV infection and processed for H&E staining. Representative photos of lungs sections showing a range of lesions consistent with early diffuse alveolar damage (DAD) were captured at 400X magnification. Hyaline membranes are indicated by the black arrows. (A) Scattered cellular sloughing, necrotic debris (inset), and increases in cellularity by immune cell infiltration were present. (B) Regions of alveolar hemorrhage adjacent to hyaline membranes are indicated by asterisks. (C) The formation of prominent hyaline membranes in multiple regions of the lung were visible. (PDF) [file ppat.1006810.s003.pdf]

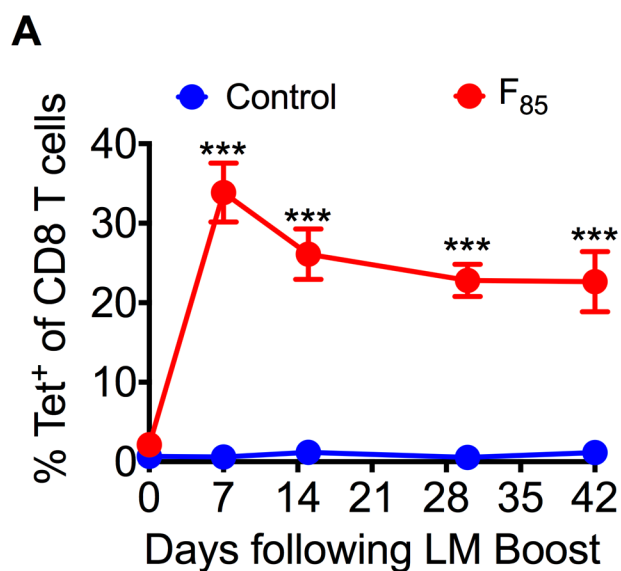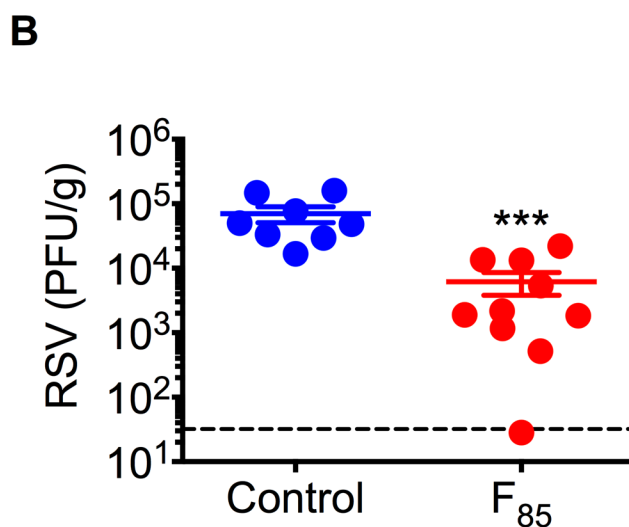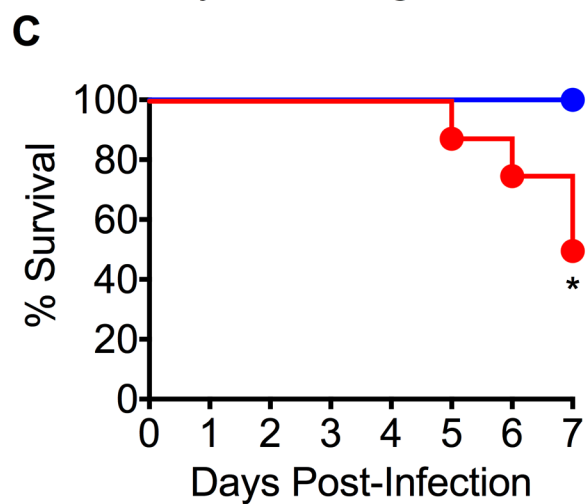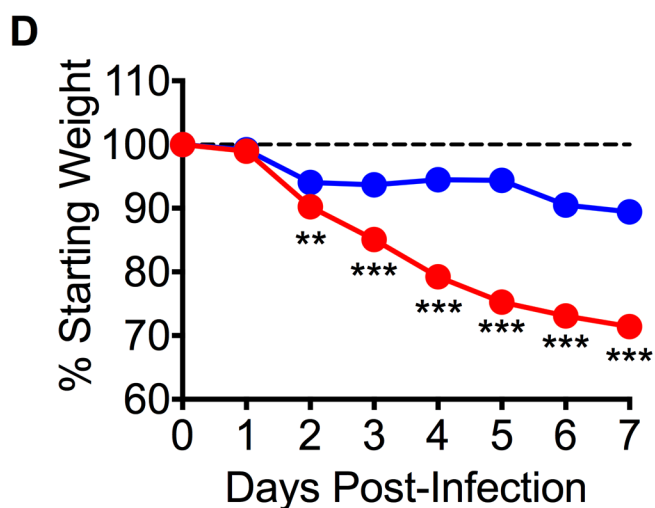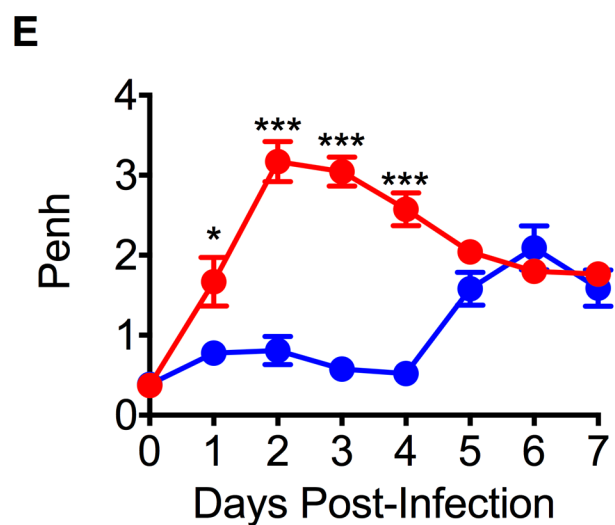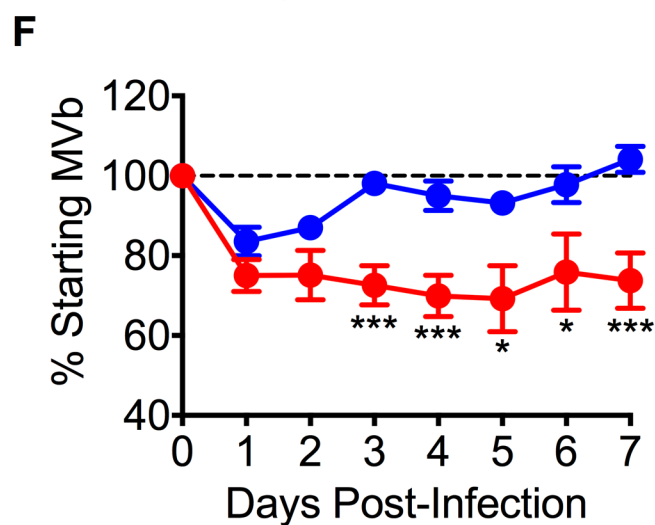

Supplement: S4 Fig — Naive BALB/c mice were control- or F85-immunized as in Fig 1 and infected with RSV 42 days later. (A) F85-tetramer+ CD8 T cell response was measured in the PBL following LM booster immunization. (B) RSV titers at day 4 p.i. were determined via plaque assay in the lung. (C) Survival, (D) weight loss, (E) Penh, and (F) MVb were evaluated daily following RSV infection. Results are presented as mean ± SEM of two independent experiments (n = 8 mice). Groups were compared using Student’s t test, * p<0.05, ** p<0.01, *** p<0.001. (PDF) [file ppat.1006810.s004.pdf]

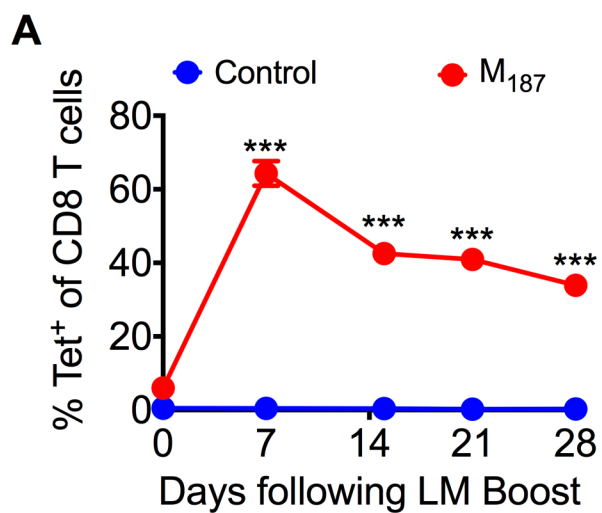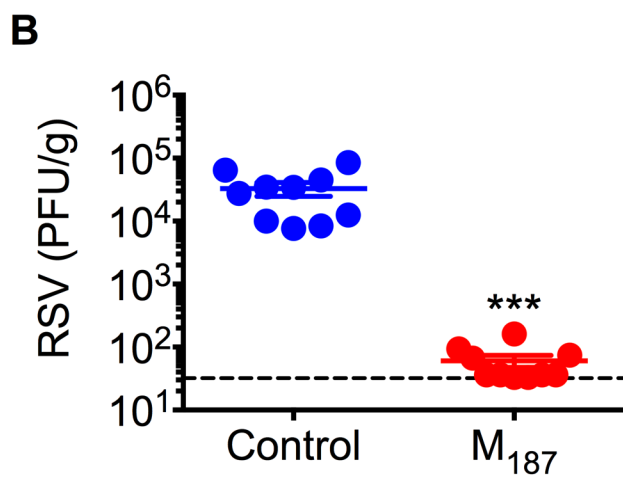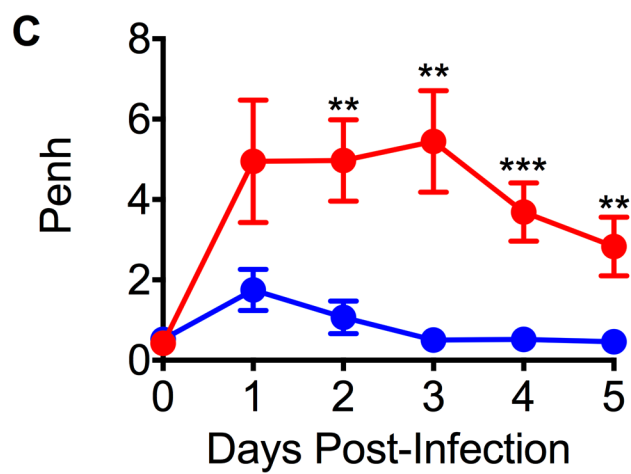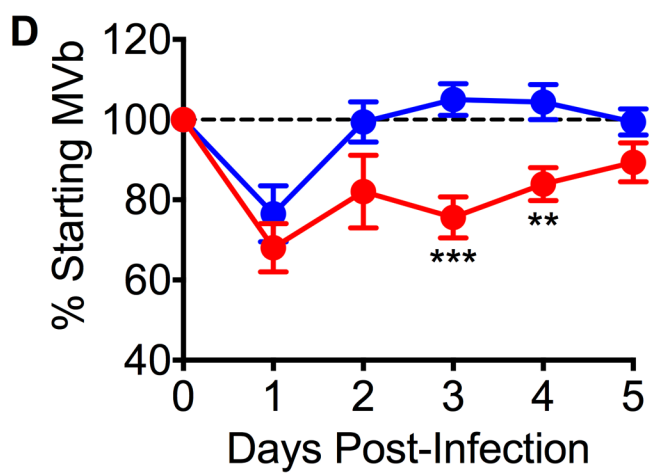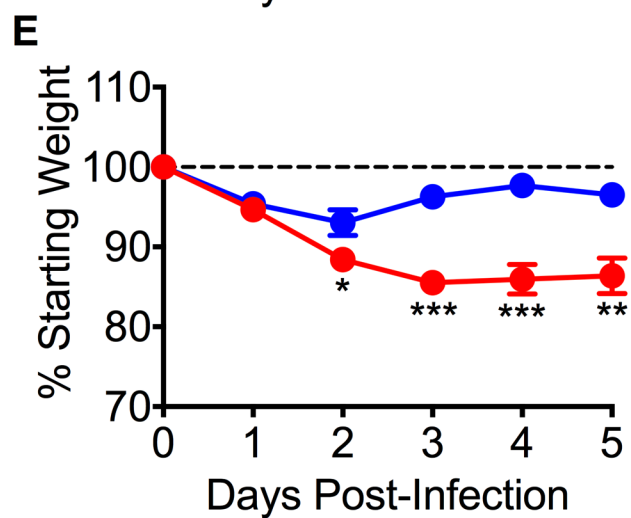

Supplement: S5 Fig — Naive C57BL/6 mice were immunized as in Fig 1, but targeting the immunodominant CD8 epitope M187. (A) Frequency of M187-specific CD8 T cells was determined by tetramer staining the PBL following the LM booster immunization. (B) RSV titers in the lung were assessed at day 4 following challenge. (C) Penh, (D) MVb, and (E) weight loss were monitored daily following RSV infection. Data are presented as mean ± SEM of two independent experiments (n = 10 mice for viral titers; n = 8 for disease assessment). Groups were compared using Student’s t test, * p<0.05, ** p<0.01,*** p<0.001. (PDF) [file ppat.1006810.s005.pdf]

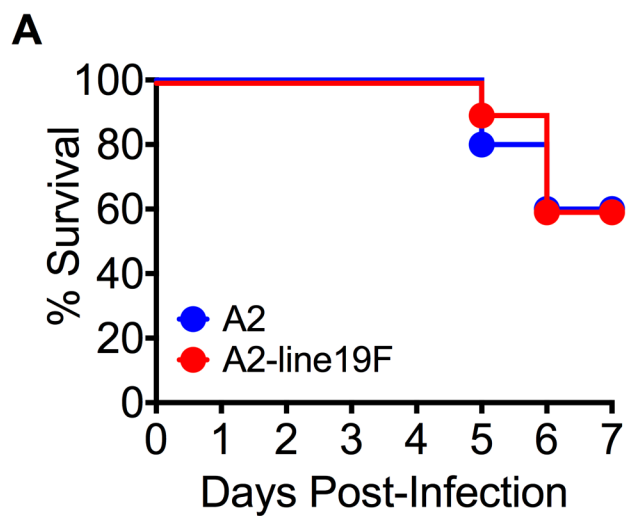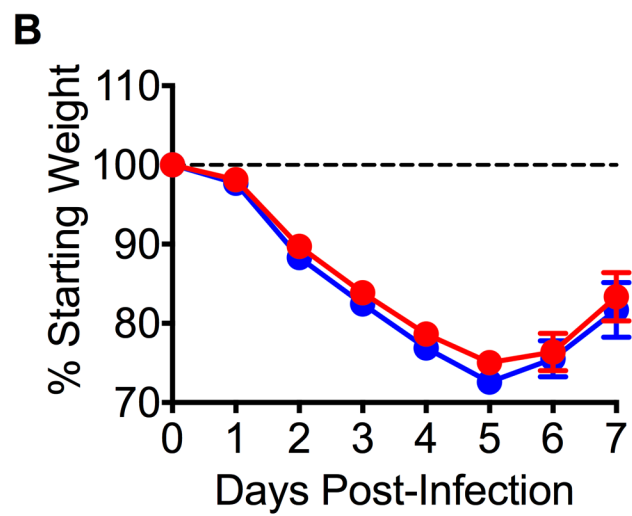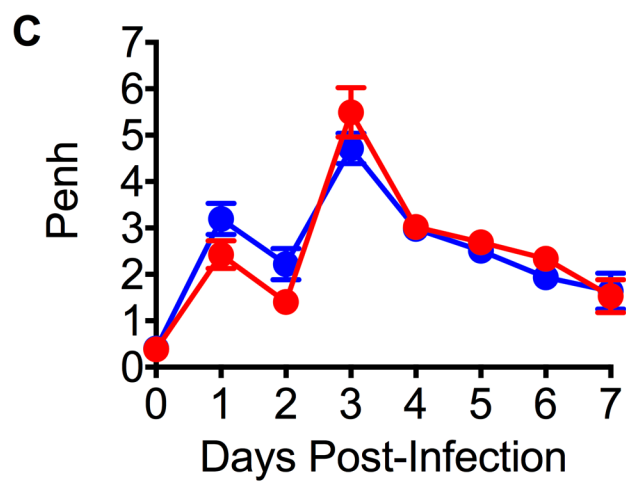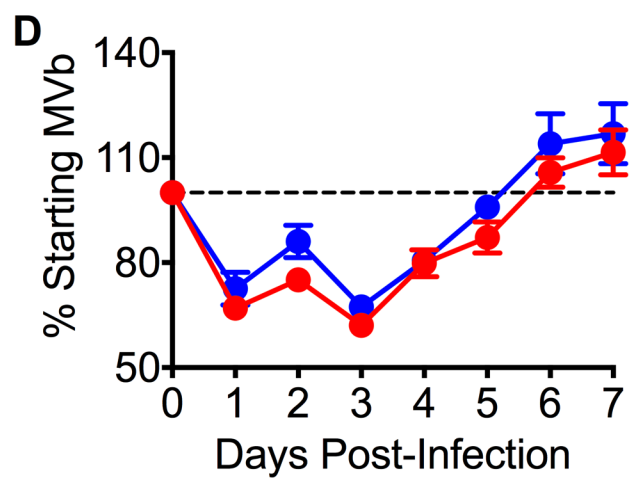

Supplement: S6 Fig — M282-immunized mice were challenged with either A2 or A2-line19F RSV strains and monitored daily for (A) survival, (B) weight loss, (C) Penh, and (D) MVb. Data are represented as mean ± SEM of two independent experiments (n = 10 mice). (PDF) [file ppat.1006810.s006.pdf]

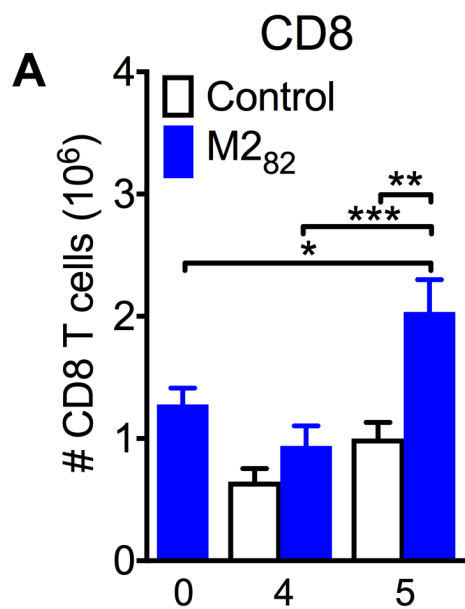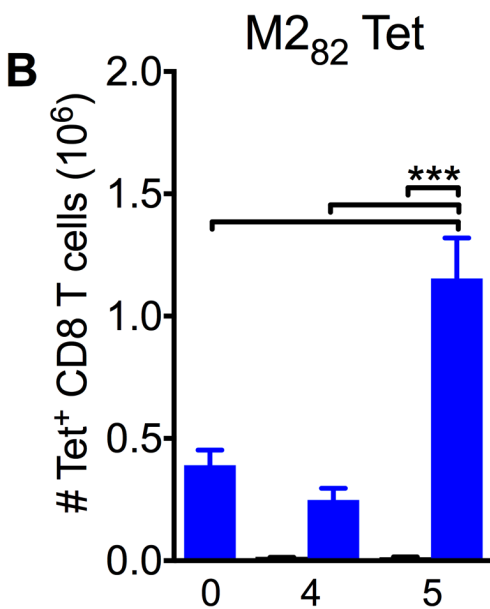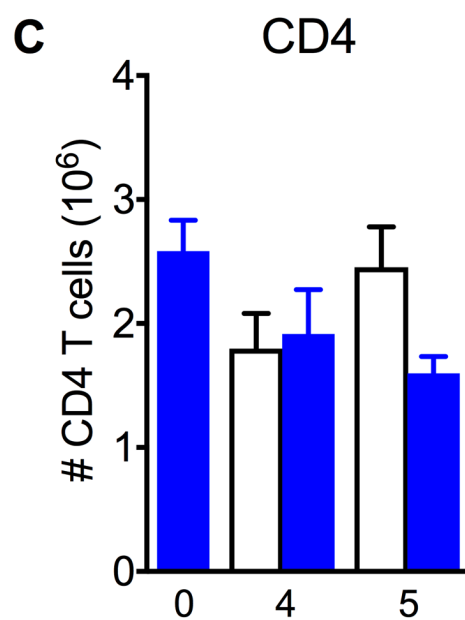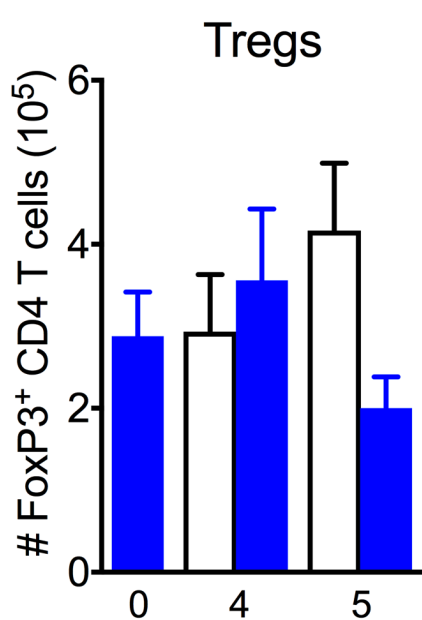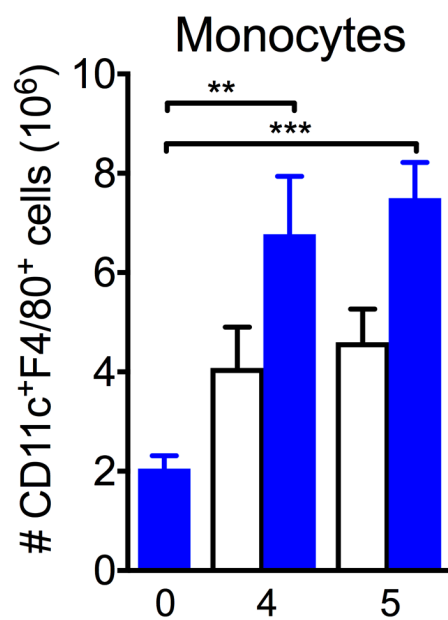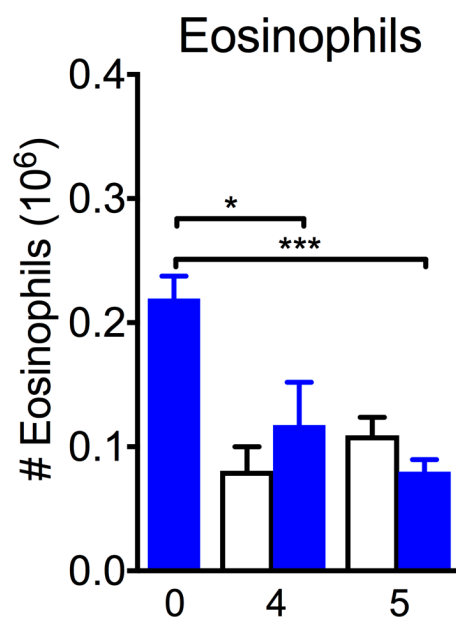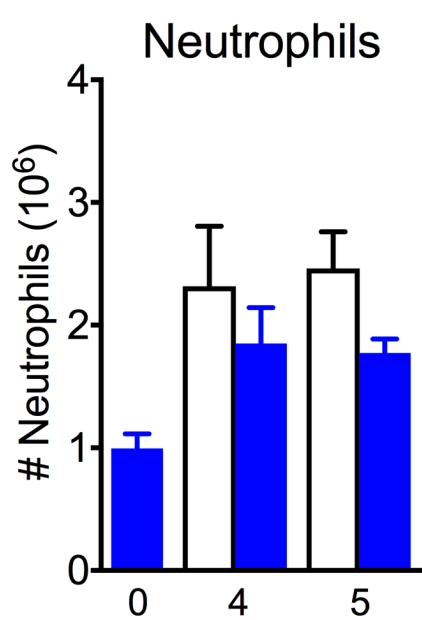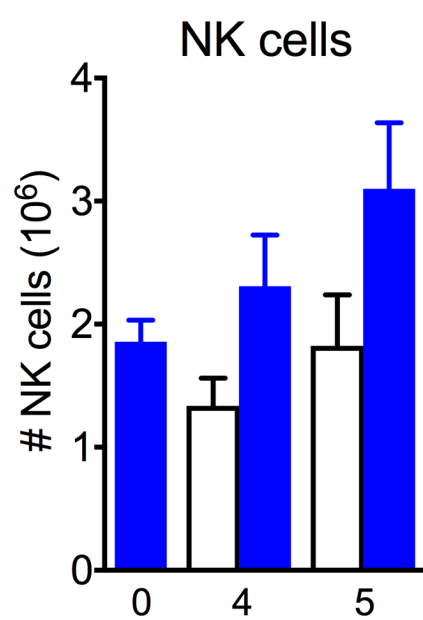

Supplement: S7 Fig — Control and M282-immunized mice were challenged with a 5 LD50 dose of IAV-M282. (A) Total CD8 and (B) M282-specific CD8 T cells in the lungs of immunized mice at days 0, 4, and 5 p.i. (C) Total numbers of CD4 T cells, Tregs, monocytes, eosinophils, neutrophils, and NK cells in the lungs on days 0, 4, and 5 p.i. Data are represented as mean ± SEM of two independent experiments (n = 8 mice). Groups within each cell type were compared using one-way ANOVA, * p<0.05, ** p<0.01, *** p<0.001. (PDF) [file ppat.1006810.s007.pdf]

**A**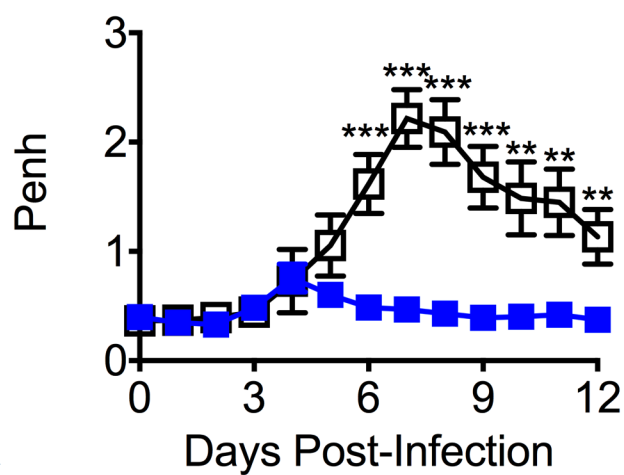**B**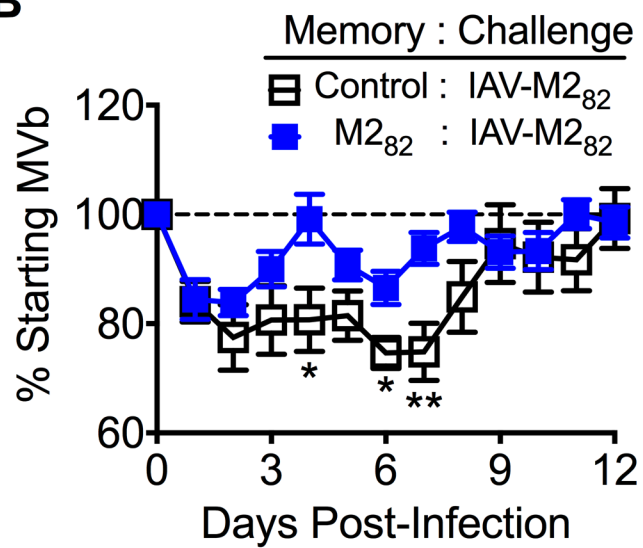**C**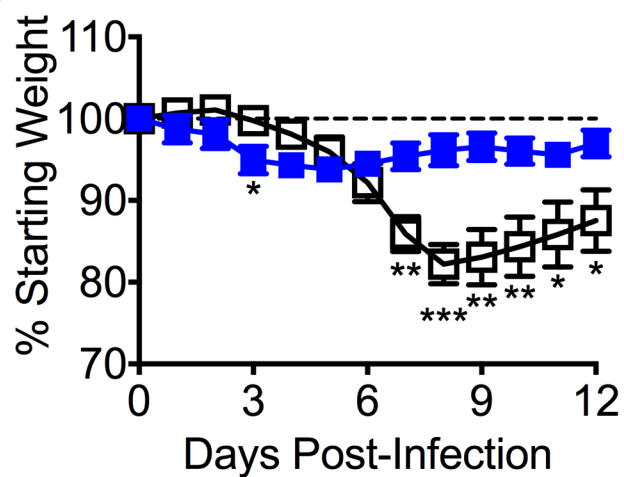

Supplement: S8 Fig — Control- and M282-immunized mice were challenged with a sublethal 0.1 LD50 dose of IAV-M282 and assessed daily for (A) Penh, (B) MVb, and (C) weight loss. Data are presented as mean ± SEM of two independent experiments (n = 8 mice for control group and n = 10 for M282 group). Groups were compared using Student’s t test, * p<0.05, ** p<0.01, *** p<0.001. (PDF) [file ppat.1006810.s008.pdf]

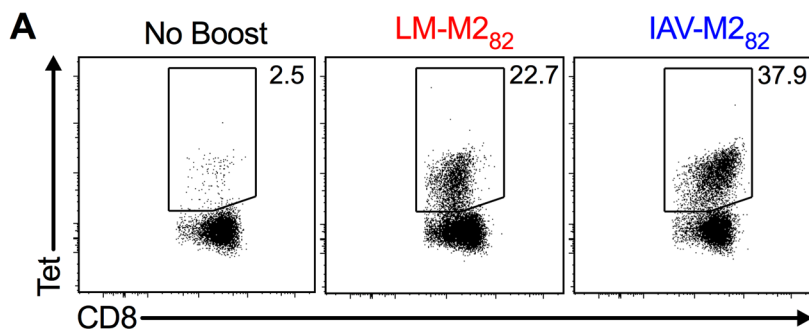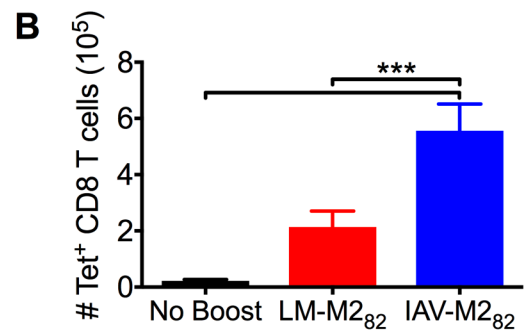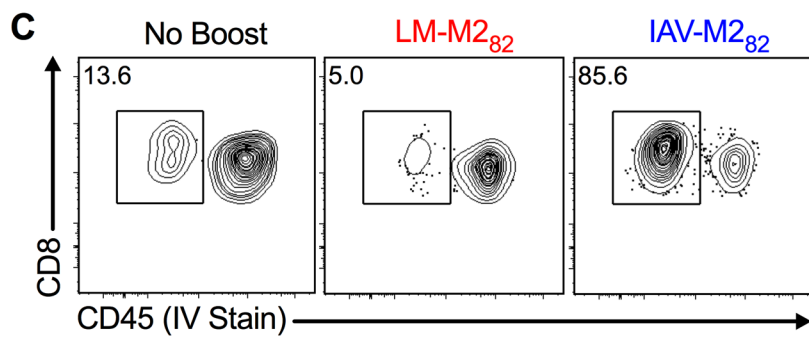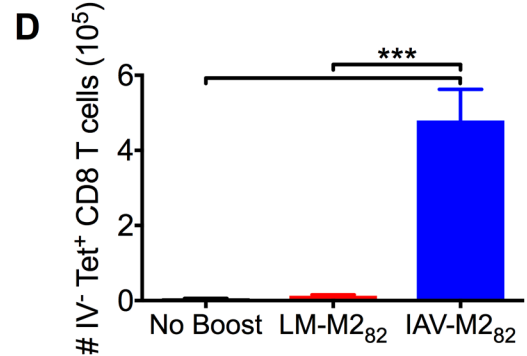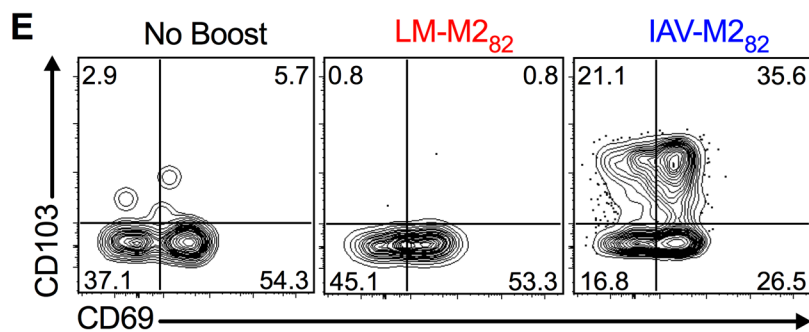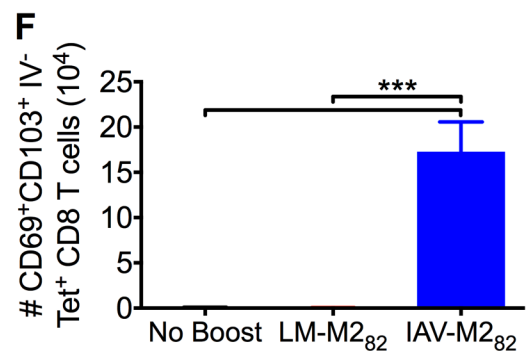

Supplement: S9 Fig — DC-M282-primed mice were either not boosted or boosted with either LM-M282 i.v. or IAV-M282 i.n. 33 days post-boost mice were administered anti-CD45 antibody i.v. 3 mins prior to harvest to stain cells within the vasculature. Representative flow plots (A) and total numbers (B) of M282-tetramer+ CD8 T cells in the lung. Representative flow plots (C) and total numbers (D) of IV stain- M282-tetramer+ CD8 T cells within the lung tissue. Representative flow plots (E) and total numbers (F) of resident memory IV stain- M282-specific CD8 T cells in the lung. Data are represented as mean ± SEM of two independent experiments (n = 8 mice). Groups were compared using one-way ANOVA, *** p<0.001. (PDF) [file ppat.1006810.s009.pdf]

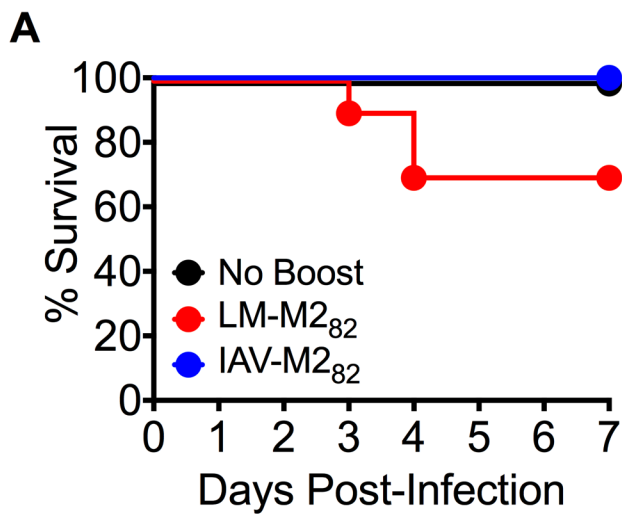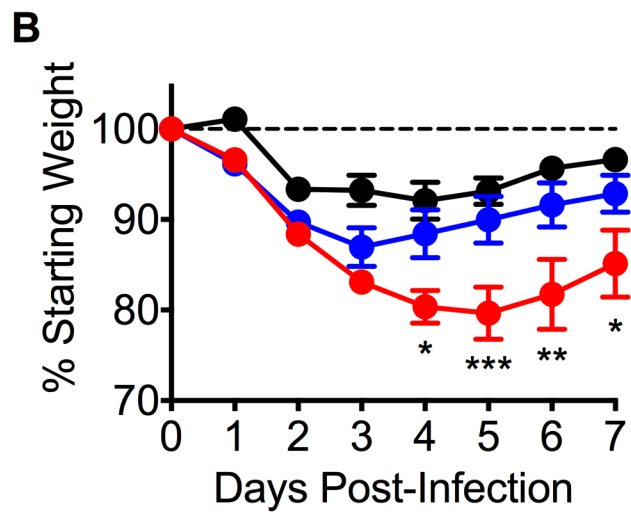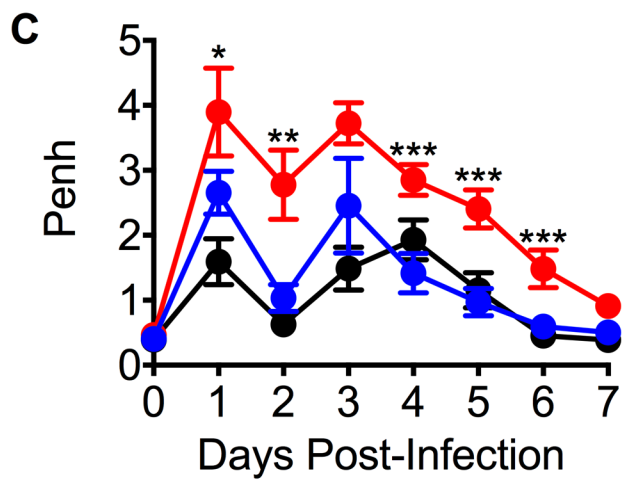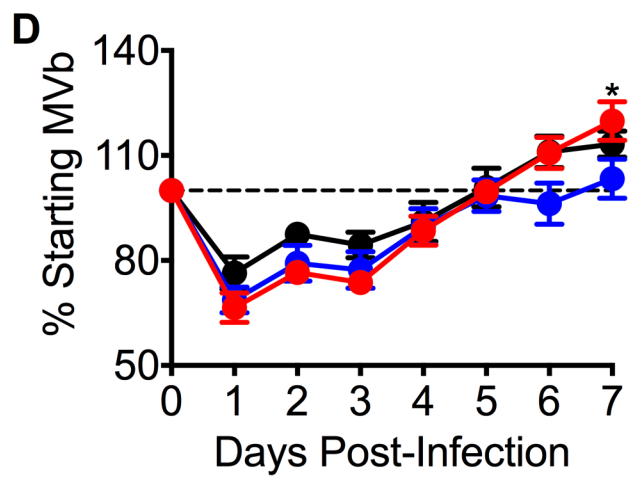

Supplement: S10 Fig — DC-M282-primed mice were either not boosted or boosted with either LM-M282 i.v. or IAV-M282 i.n. Mice were assessed for (A) survival, (B) weight loss, (C) Penh, and (D) MVb following RSV infection. Data are represented as mean ± SEM of two independent experiments (n = 10 mice). Groups were compared using one-way ANOVA, * p<0.05, ** p<0.01, *** p<0.001. Asterisks represent statistical significance between LM-M282 and IAV-M282 groups. (PDF) [file ppat.1006810.s010.pdf]

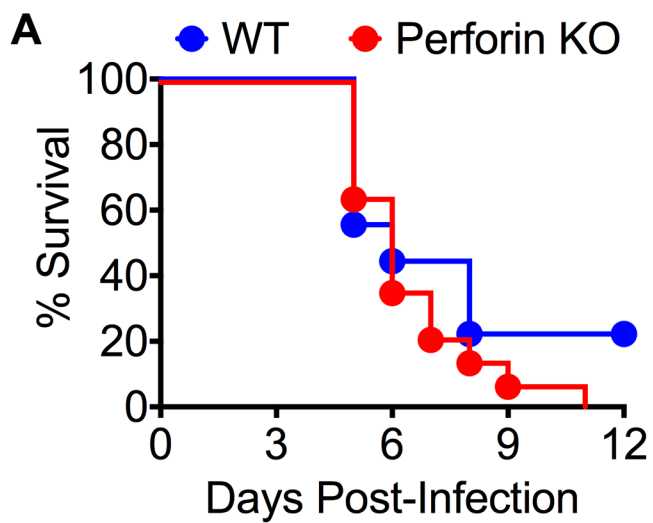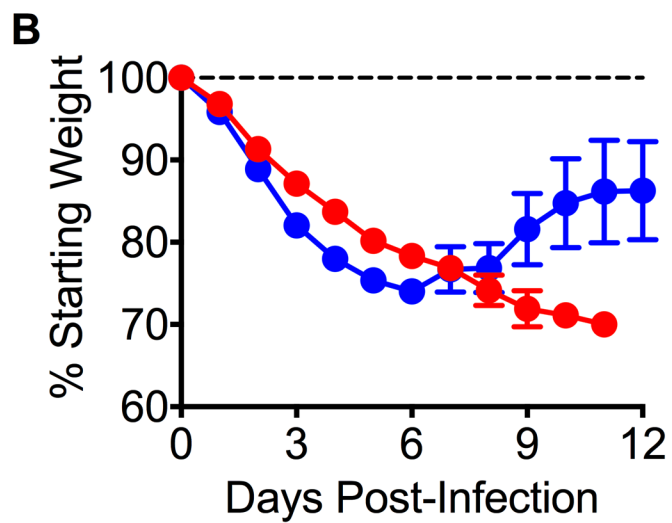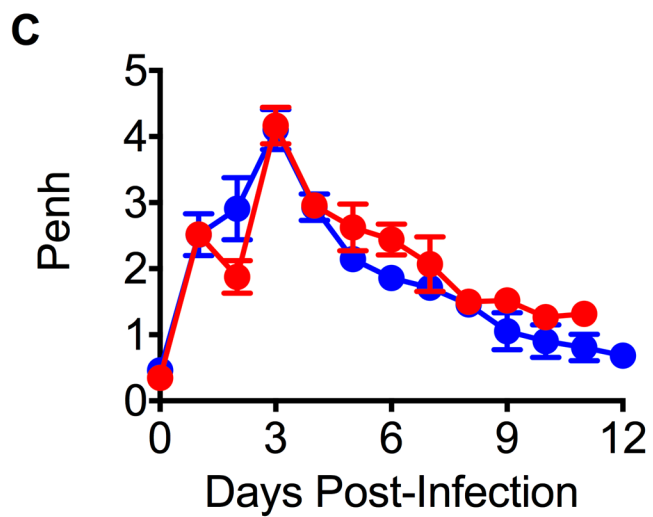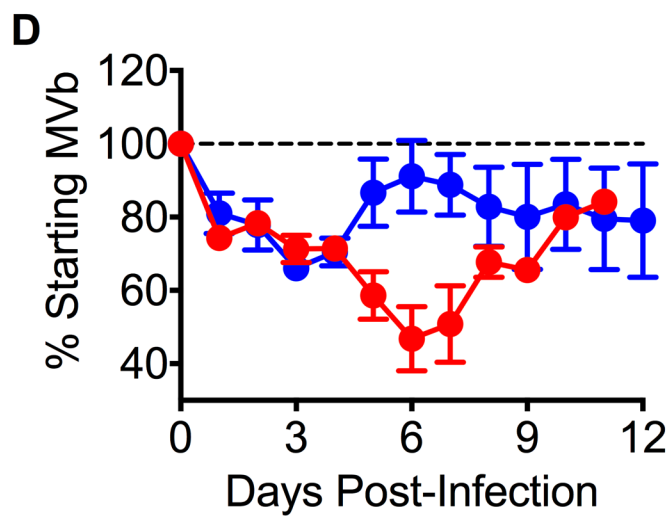

Supplement: S11 Fig — M282 DC-LM-immunized WT and perforin knock out (KO) mice were challenged with RSV 28 days later and monitored daily for (A) survival, (B) weight loss, (C) Penh, and (D) MVb. Data are presented as mean ± SEM of two independent experiments (n = 11 WT; n = 14 perforin KO). (PDF) [file ppat.1006810.s011.pdf]

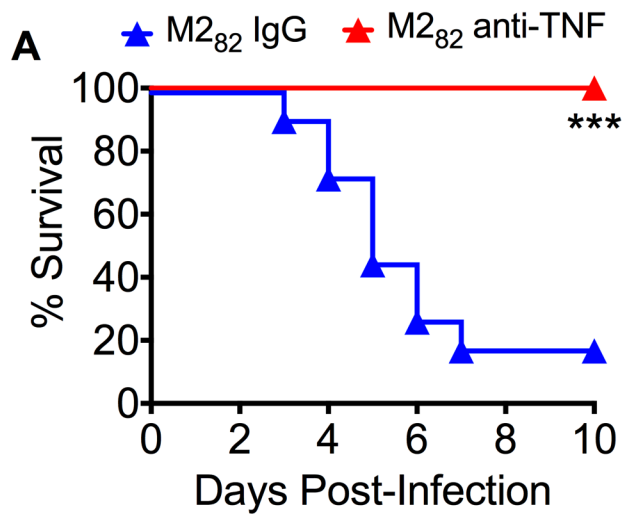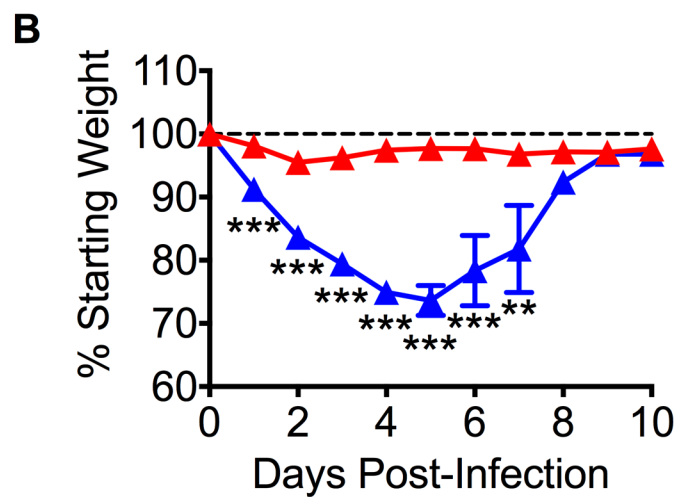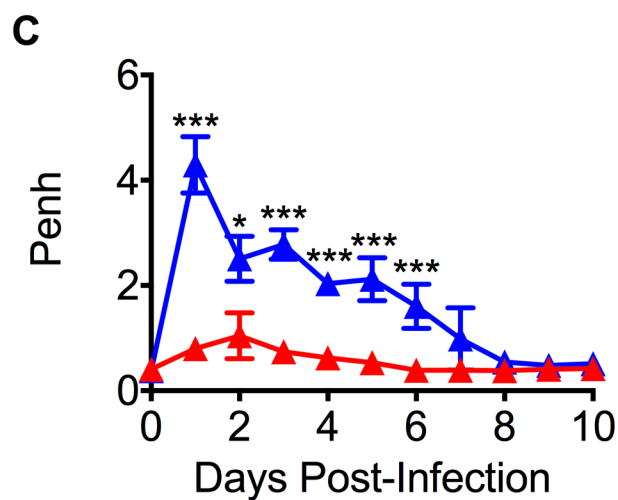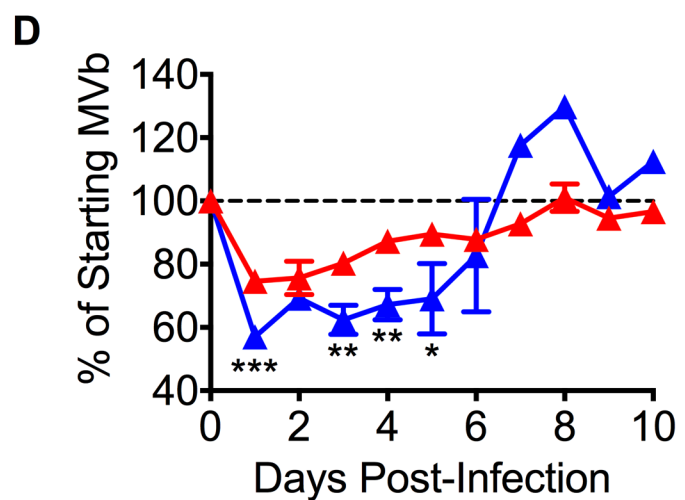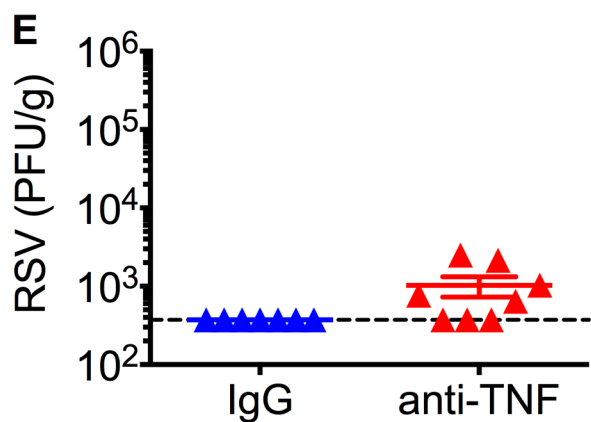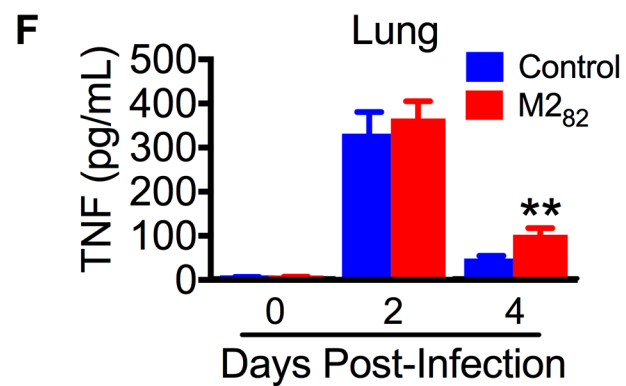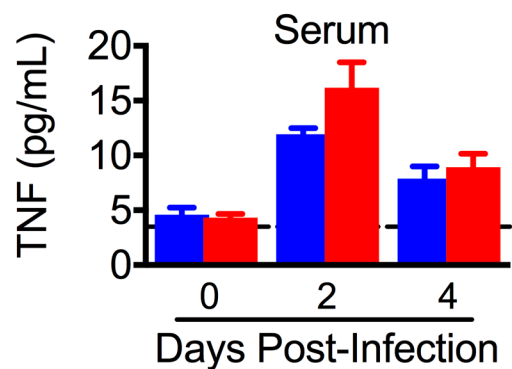

Supplement: S12 Fig — M282-immunized mice were treated with 200 μg of either IgG or anti-TNF antibody i.n. during the time of RSV infection. (A) Survival, (B) weight loss, (C) Penh, and (D) MVb were assessed daily following RSV challenge. (E) RSV titers in the lung were determined via plaque assay at day 4 p.i. (F) TNF protein amounts were quantified at days 0, 2, and 4 p.i. in the lung and serum of control- and M282-immunized mice. Data are presented as mean ± SEM of two independent experiments (n = 11 in (A-D); n = 8 in (E); n = 6 for control and n = 8 for M282 in (F)). Statistical comparisons were performed using Student’s t test, * p<0.05, ** p<0.01, *** p<0.001. (PDF) [file ppat.1006810.s012.pdf]

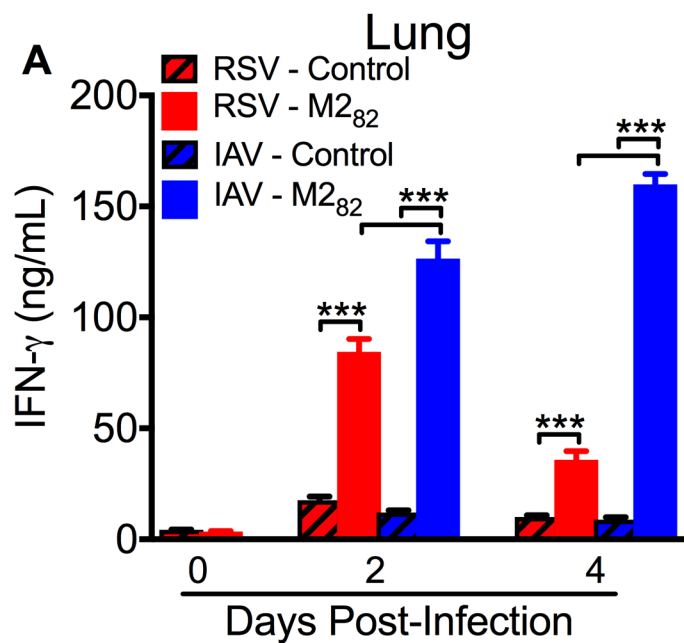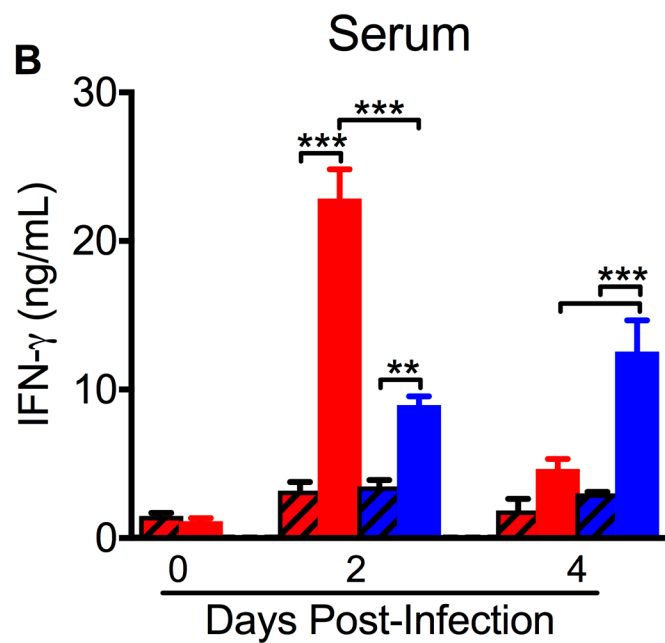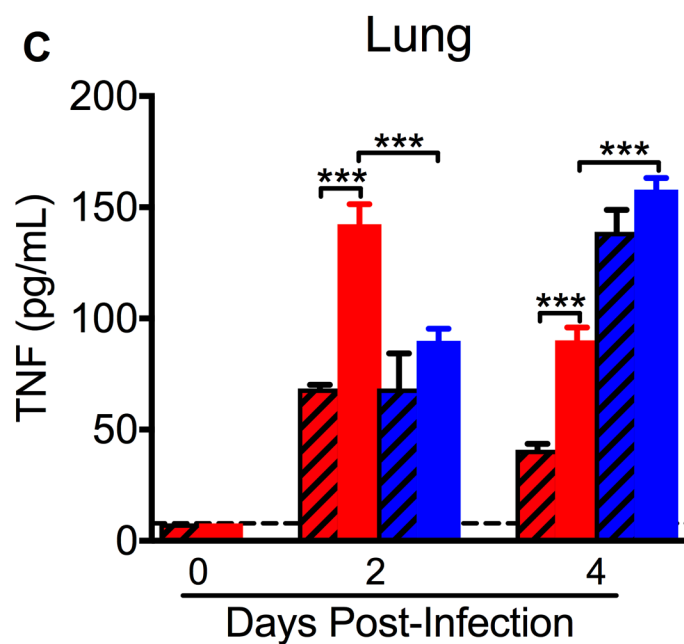

Supplement: S13 Fig — Control and M282-immunized mice were challenged with either RSV or a 5 LD50 dose of IAV-M282. IFN-γ protein levels were determined in the (A) lung and (B) serum at 0, 2, and 4 days p.i. by ELISA. (C) TNF protein levels were quantified in the lung at 0, 2, and 4 days p.i. by ELISA. Data are represented as mean ± SEM of two independent experiments (n = 8 mice). Groups were compared using one-way ANOVA, * p<0.05, ** p<0.01, *** p<0.001. (PDF) [file ppat.1006810.s013.pdf]

**A**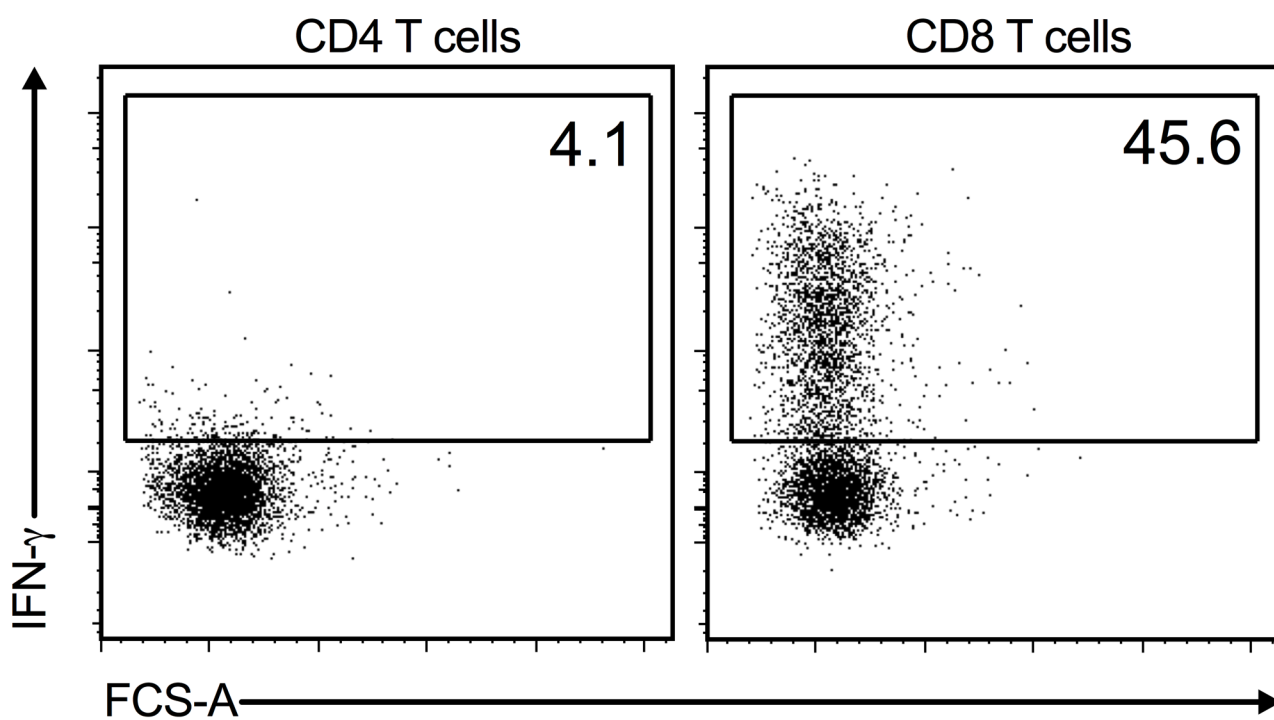**B**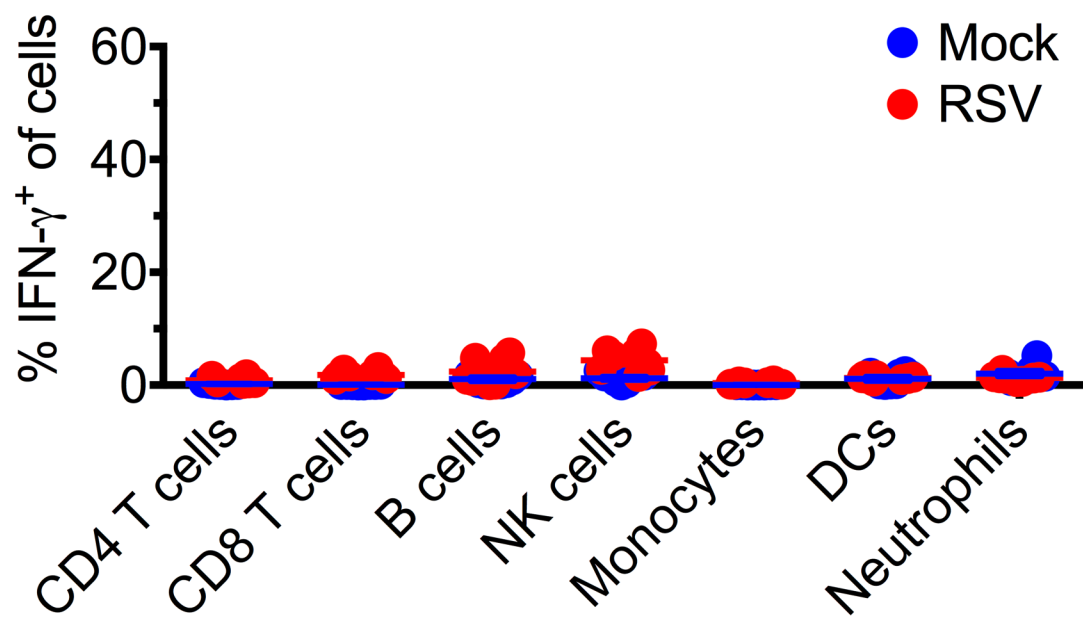

Supplement: S14 Fig — DC-LM-immunized mice were administered 250 μg BFA i.v. 6 hours prior to organ collection. (A) Representative flow plots of IFN-γ production gated on CD4 and CD8 T cells at day 2 p.i. in the lung. (B) IFN-γ secretion by leukocyte populations in the lung at day 5 following either mock or RSV challenge. Data are presented as mean ± SEM of two independent experiments (n = 8 mice). (PDF) [file ppat.1006810.s014.pdf]
